# HLA-B27 detection test for individuals with suspected axial spondyloarthritis to Brazilian public health system: accuracy, cost-effectiveness, and budget impact analysis

**DOI:** 10.3205/hta000141

**Published:** 2026-07-01

**Authors:** Vinicius Lins Ferreira, Layssa Andrade Oliveira, Haliton Oliveira Junior, Rosa Camila Lucchetta

**Affiliations:** 1 Health Technology Assessment Unit, Hospital Alemão Oswaldo Cruz, São Paulo, São Paulo, Brazil

**Keywords:** spondyloarthritis, Brazil, diagnosis, HLA-B27 antigen

## Abstract

**Objective:**

the aim of this study is to assess the accuracy, cost-effectiveness, and budget impact of the HLA-B27 test in diagnosis of axial spondyloarthritis to Brazilian Public Health System (SUS).

**Methods:**

A systematic review was conducted to assess the sensitivity and specificity of HLA-B27 compared to test ASAS or New York diagnostic criteria. Additionally, a cost-effectiveness analysis (a decision tree model coupled with a Markov model and effectiveness outcome of quality-adjusted life year (QALY)) and budget impact analysis were also developed. Costs were obtained in Brazilian currency and converted to dollars considering the PPP conversion factor.

**Results:**

Twenty-nine studies have shown that the sensitivity of the HLA-B27 test was 68% (95% CI: 67-69%) and the specificity was 88% (95% CI: 87-88%) (very low certainty of evidence to both outcomes) when compared with any comparator. The analysis of two studies showed that the combination of HLA-B27+clinical parameters still achieved sensitivity and specificity comparable to or greater than the combination of imaging and clinical parameters, or clinical parameters alone. When compared with clinical evaluation and clinical evaluation±imaging, the HLA-B27 test+clinical evaluation was close to the cost-effectiveness threshold ($17,628 per QALY) and dominated the comparator, respectively. Furthermore, incorporating HLA-B27 into the SUS would result in an increase in costs of $530,393 in 5-years of analysis (mean of six thousands/year eligible patients to HLA-B27).

**Conclusion:**

The findings supported the coverage of the HLA-B27 test in SUS, considering that test increases accuracy when associated with other diagnostic strategies and has the potential to be cost-effective or dominant.

## Zusammenfassung

**Zielsetzung:**

Ziel dieser Studie ist es, die Genauigkeit, Kosteneffektivität und Budgetauswirkungen des HLA-B27-Tests bei der Diagnose von axialer Spondyloarthritis im brasilianischen öffentlichen Gesundheitssystem (SUS) zu bewerten.

**Methoden:**

Es wurde eine systematische Übersichtsarbeit durchgeführt, um die Sensitivität und Spezifität von HLA-B27 im Vergleich zu den ASAS- oder New-York-Diagnosekriterien zu ermitteln. Darüber hinaus wurden eine Kosten-Nutzen-Analyse (ein Entscheidungsbaum-Modell in Kombination mit einem Markov-Modell und dem Effektivitätsmaß Quality-Adjusted Life Year, QALY) sowie eine Budget-Impact-Analyse erstellt. Die Kosten wurden in brasilianischer Währung erhoben und unter Berücksichtigung des Kaufkraftparitäten-Faktors (PPP) in US-Dollar umgerechnet.

**Ergebnisse:**

Neunundzwanzig Studien zeigten, dass die Sensitivität des HLA-B27-Tests 68 % (95%-KI: 67-69 %) und die Spezifität 88 % (95%-KI: 87-88 %) betrug (sehr geringe Evidenzsicherheit für beide Endpunkte), wenn der Test mit beliebigen Vergleichsmethoden gegenübergestellt wurde. Die Analyse zweier Studien ergab, dass die Kombination aus HLA-B27 und klinischen Parametern eine Sensitivität und Spezifität erreichte, die vergleichbar oder höher war als die Kombination aus Bildgebung und klinischen Parametern oder nur klinischen Parametern. Im Vergleich zur klinischen Untersuchung bzw. zur klinischen Untersuchung ± Bildgebung lag die Kombination aus HLA-B27-Test und klinischer Untersuchung nahe an der Kosteneffektivitätsgrenze (17.628 $ pro QALY) bzw. dominierte den Vergleich. Darüber hinaus würde die Einführung von HLA-B27 in das SUS innerhalb eines Analysezeitraums von fünf Jahren zu einer Kostensteigerung von 530.393 $ führen (im Mittel etwa sechstausend pro Jahr für den Test infrage kommende Patienten).

**Schlussfolgerung:**

Die Ergebnisse unterstützen die Aufnahme des HLA-B27-Tests in das SUS, da der Test in Kombination mit anderen diagnostischen Strategien die Genauigkeit erhöht und potenziell kosteneffektiv oder sogar überlegen sein kann.

## Introduction

Axial spondyloarthritis (SpA) is a chronic inflammatory autoimmune disease that primarily affects the spine, but also peripheral joints, and extra-articular sites such as the eye and intestine. It is characterized by intense pain, joint stiffeness due to enthesitis (inflammation at tendon insertions), and progressive functional impairment, leading to irreversible structural damage to the sacroiliac and spinal joints, accompanied by radiographic changes and excessive bone formation. Progression of the arthropathy can lead to spinal fusion and cause extreme disability, with loss of mobility in the spine, pelvis, and lower back [1], [2], [3], [4], [5].

SpA affects approximately 0.1 to 1.5% of the population, with significant geographic variation in prevalence and main clinical and phenotypic manifestations [6], [7], [8], [9]. A study reported the prevalence of the disease in South America as 0.14% (95% CI 0.02%–0.34%) [10].

The HLA-B27 antigen is strongly correlated with the onset of the disease, and a positive test for this marker is found in 80% to 98% of cases [11], [12]. In Brazil, several studies suggest smaller proportions, where HLA-B27 antigen carriers represent approximately 60–70% of patients [13], [14], [15], [16], [17], [18], [19], [20], [21]. 

The ASAS (Assessment of SpondyloArthritis International Society) criteria allow the diagnosis of patients without structural damage, and the modified New York classification criteria allow the diagnosis of patients with radiographic changes, in a more advanced stage of the disease. ASAS classification criteria for SpA (patients with back pain ≥3 months and age of onset <45 years) should be one of the following:

1. Sacroiliitis on imaging + ≥1 SpA feature, or
2. HLA-B27 + ≥2 others SpA features (note: SpA features includes inflammatory back pain, arthritis, enthesitis, uveitis, dactylitis, Crohn disease/ulcerative colitis, psoriasis, family history of spondyloarthritis, good response to nonsteroidal anti-inflammatory drugs, HLA-B27 positivity, elevated C-reactive protein). However, the ASAS diagnostic criteria may include the HLA-B27 test, which is not available in the Brazilian public health system [22].

In Brazilian public health system (i.e., SUS; Brazil’s Unified Health System), the process of decision about coverage of technologies involves requests based on evidence of efficacy, effectiveness, accuracy, safety, cost-effectiveness and budgetary impact for the system [23]. In this context, the objective of this study is to report the potential impact of inclusion of HLA-B27 test in diagnosis of individuals with suspected axial spondyloarthritis into SUS.

## Methods

### Systematic review

Initially, an updated of a systematic review originally conducted by the National Institute for Health and Care Excellence (NICE; published in 2016) was done [24]. For guide this update, the following research question was formulated: “in individuals with suspected axial spondyloarthritis, what is the sensitivity, specificity, and diagnostic utility of the HLA-B27 test compared to the ASAS criteria, New York criteria and diagnosis by a specialist”? The PIRO question is presented below (Table 1 (Tab. 1)). 

Based on the PIRO question presented before, a search was conducted in January 2023 on the PubMed, Cochrane Library, and EMBASE platforms (see Tab. S1 in Attachment 1). This systematic review was conducted according to Cochrane handbook to diagnostic test accuracy [25], and reported according to PRISMA recommendations [26]. 

The retrieved records were imported into Rayyan [27], where duplicates were identified and removed. Then, the records were selected by a single reviewer, with a second reviewer consulted for screening (reading titles and abstracts) and eligibility (reading full texts) when necessary.

Data extraction was performed by a single evaluator using Microsoft Office Excel^®^ spreadsheets. The following data were extracted:

- Study and participant characteristics: author, year, country, general characteristics of the population (age, sex, disease duration), number of participants, alternatives compared, reference test, and inclusion criteria.
- Diagnostic accuracy outcomes: Sensitivity, specificity, and absolute numbers (true positives, true negatives, false positives, and false negatives) extracted directly from the text. When unavailable, sensitivity and specificity were calculated from absolute numbers.

To assess the risk of bias in the studies, the Quality Assessment of Diagnostic Accuracy Studies (QUADAS-2) tool was used [28]. The quality or confidence of the evidence was assessed considering the GRADE (Grading of Recommendations Assessment, Development and Evaluation) approach for diagnostic accuracy studies [29], [30], [31]. 

Whenever possible, individual results were pooled in meta-analyses conducted in Meta-DiSc (version 1.4) [32]. Sensitivity, specificity, and predictive values were calculated with a 95% confidence interval, and the sROC (receiver operator characteristic curve) and the area under the curve (AUC) were established based on these. Subgroup analyses were performed considering the different comparators:

1. ASAS diagnostic criteria without HLA-B27,
2. modified New York diagnostic criteria, and
3. other diagnostic criteria (rheumatologists, more than one diagnostic criterion evaluated in the same study, or other criteria). Heterogeneity test, such as I2 statistics, was not performed, as it is not recommended for systematic reviews of test accuracy [33].

### Economic analysis

An economic evaluation was conducted to estimate the incremental cost-effectiveness ratio (ICER) of diagnosis strategies for patients with a suspected diagnosis of axial spondyloarthritis, adhering to the CHEERS Task Force Report – 2022 reporting checklist [34]. The age at entry into the model was 35 years old, with an equal sex distribution (50% male, 50% female), reflecting the average demographics of the studies included in the clinical evidence synthesis [35], [36], [37], [38], [39], [40]. The perspective adopted was SUS.

The intervention analyzed was the HLA-B27 testing and clinical evaluation, defined as at least two features of SpA (i.e., inflammatory back pain according to specialists; extraspinal manifestations such as arthritis, enthesitis, uveitis, psoriasis, ulcerative colitis, or Crohn’s disease; good response to nonsteroidal anti-inflammatory drugs; family history of SpA; elevation in C-reactive protein or erythrocyte sedimentation rate).

The comparators defined for this analysis were:

1. Clinical evaluation only: three or more SpA characteristics; or
2. Clinical evaluation ± imaging exam: at least 1 SpA characteristic + sacroiliitis on imaging exam (plain X-ray or MRI); for both comparators available SpA characteristics were according to ASAS classification criteria for SpA (as described in the last paragraph).

**Figure 1:**
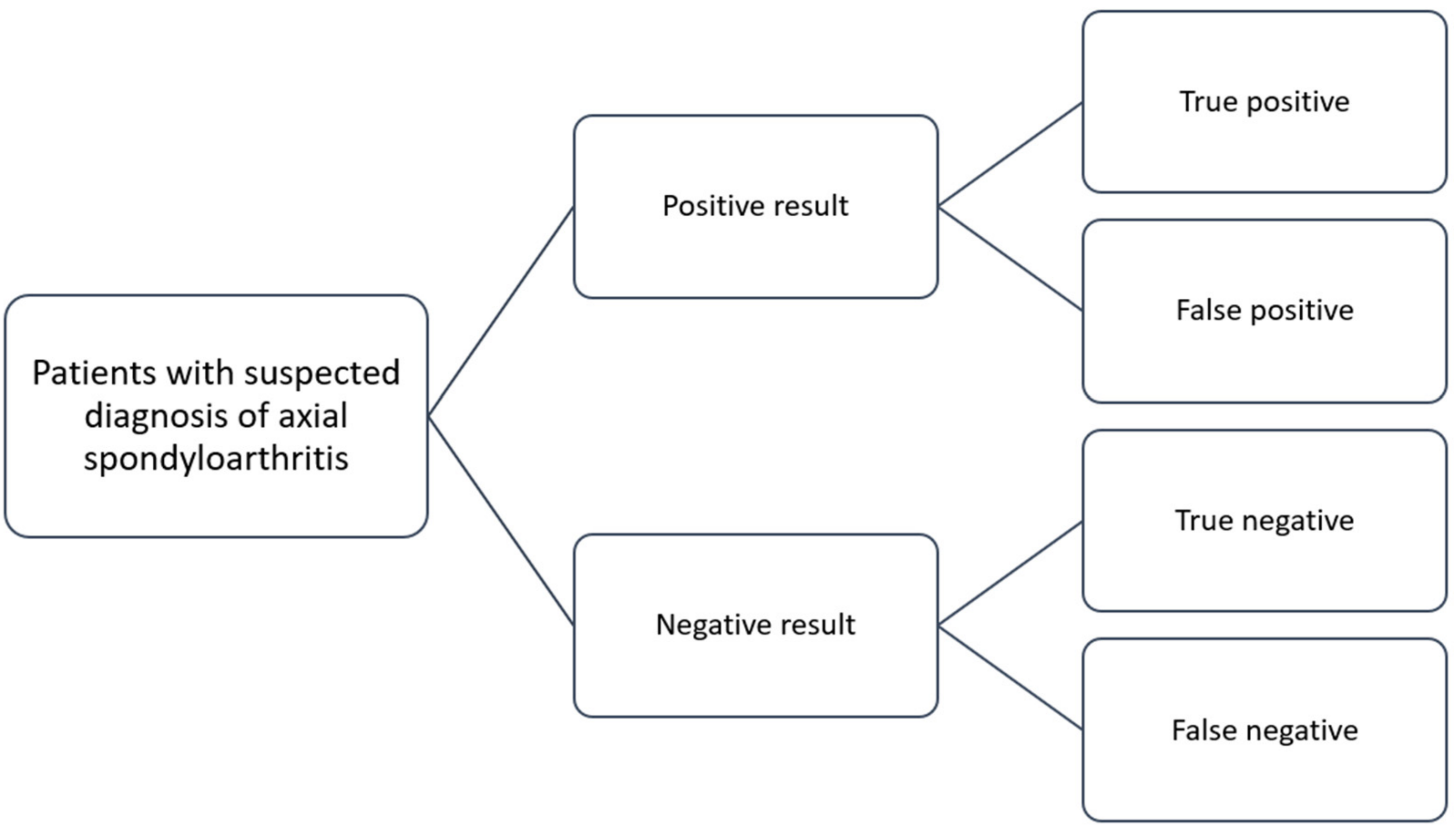
Decision tree model

A decision tree coupled with the transitional state (Markov) model was developed (Figure 1 (Fig. 1)). The decision tree represented the performance of the tests, so that a positive or negative detection result could be obtained, and these could be true or false.

**Figure 2:**
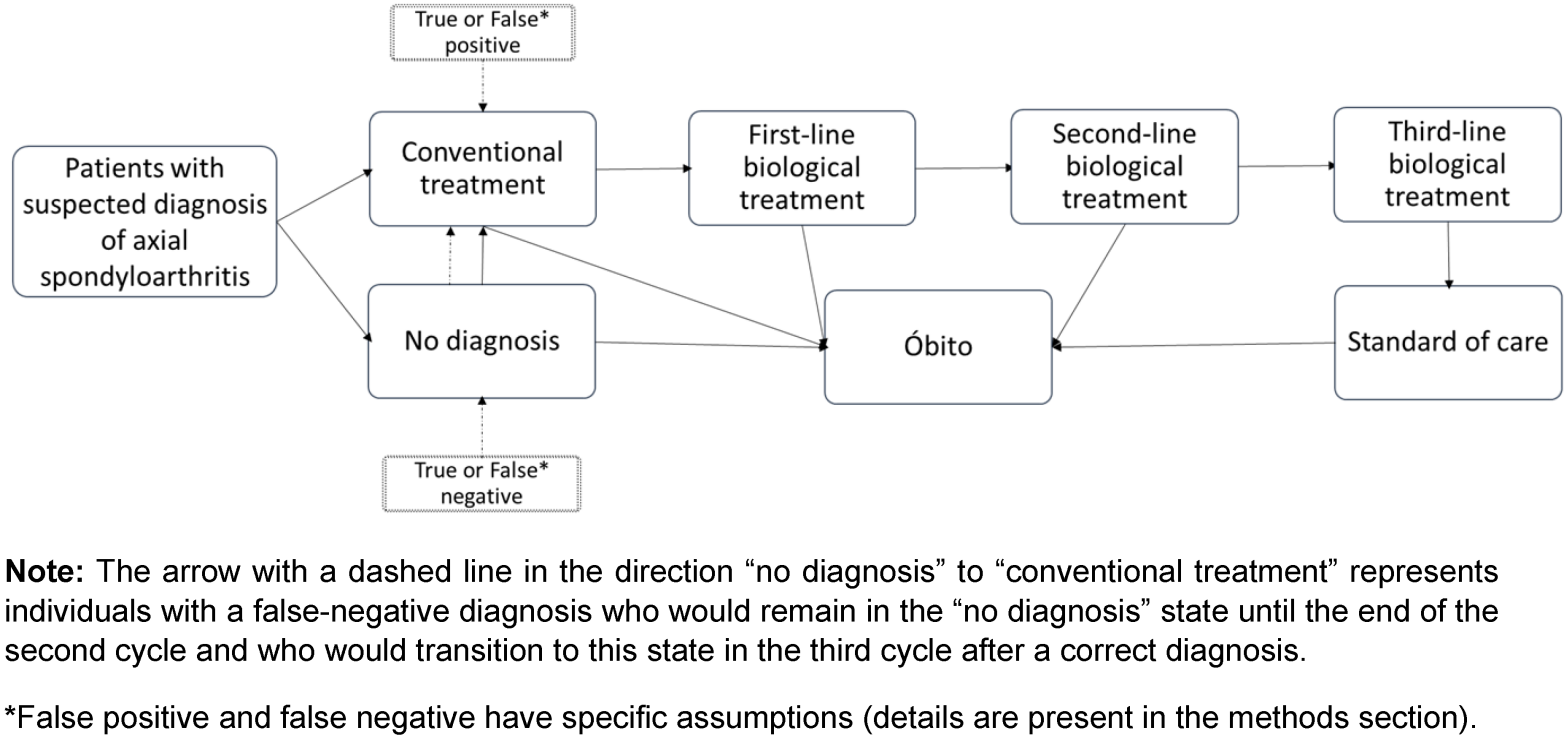
Markov model

The Markov model considered annual cycles, a 20-year time horizon, and included 7 health states, which considered disease progression: no diagnosis, treatment with conventional drugs, treatment with first-, second-, and third-line biological drugs, standard of care, and death (shown in the Figure 2 (Fig. 2)).

It was assumed that:

- Individuals with a true or false diagnosis of axial spondyloarthritis transition to the "conventional treatment" state (i.e., sulfasalazine, methotrexate or naproxen). However, individuals with a true diagnosis have a probability >0 of migrating to the other states (i.e., treatment with biological treatment with adalimumab, secukinumab, etanercept, infliximab, golimumab, or certolizumab), while individuals with a false-positive diagnosis remain in this state or transition to death;
- Individuals with a true-negative diagnosis remain in the "no diagnosis" state until the end of the model or transition to death;
- Individuals with a false-negative diagnosis remain in the "no diagnosis" state until the end of the second cycle. However, in the third cycle, they receive the correct diagnosis and transition to the other states in the following cycles. This assumption was made because it was considered that the lack of adequate treatment would result in the emergence of new disease characteristics, allowing for the correct diagnosis of the condition as the disease progresses.

The parameters reported by Rudwaleit, 2009a and Rudwaleit, 2009b were considered for this economic evaluation [22], [41]. Values related to the diagnostic accuracy of the compared strategies and other parameters are presented in Tab. S7 and S8 in Attachment 1.

The model considered only direct costs related to diagnostic procedures and treatments specific to each cycle. Costs were obtained in reais (R$), the Brazilian currency, and converted to dollars considering the PPP conversion factor (year 2023: R$ 2.45=$ 1 [42]). The main costs considered are summarized in Table 2 (Tab. 2) and more details are presented in Tab. S9 to S13 in Attachment 1.

Total direct costs and effectiveness in terms of quality-adjusted life years and correctly diagnosed individuals were considered as outcomes of this analysis (cost-effectiveness threshold $16.400 per QALY gain). A discount rate of 0.05 (5.0%) has been applied. 

A sensitivity analysis was performed to assess the uncertainties related to the estimates of the adopted parameters, as well as the reliability and robustness of the cost-effectiveness analysis. The minimum and maximum values used were previously described here and in Tab. 3 in Attachment 1. A probabilistic analysis with 1,000 iterations was performed and expressed as a scatterplot (cost data were varied according to the gamma distribution, while probability data were varied according to the beta distribution).

### Budget impact analysis

In the reference scenario, adopting SUS perspective with a five-year time horizon, the ASAS classification criteria without the HLA-B27 test were considered as an alternative available in. Therefore, the system only provides clinical evaluation or, clinical evaluation combined with the identification of sacroiliitis on imaging tests – plain radiography or magnetic resonance imaging. In the alternative scenario, the maintenance of the ASAS Classification criteria with a diagnostic evaluation strategy, with and without HLA-B27 testing, was considered.

The market share for the reference scenario was established based on scientific literature [43], data from DataSUS (Outpatient Information System - outpatient production, SIA-PA), and expert opinion. The market share for the proposed scenario considered this information and a simulated HLA-B27 test distribution of 20% in the first year, reaching 100% in the fifth year of analysis (20% increase per year).

To estimate the eligible population, measured demand was initially considered, with data obtained from the Open Health Intelligence Situation Room (SABEIS [44]). This analysis used the number of new users using medications for the treatment of ankylosing spondylitis per year (2017 to 2022). For the remaining years of the analysis (2023 to 2027), the number of patients was calculated based on the trend in users estimated from these available data.

Furthermore, the positivity of the disease in the diagnostic evaluation identified in the studies included in the systematic review was considered. For this purpose, it was assumed that for every two individuals evaluated for axial spondyloarthritis, one would be positive; in other words, twice as many individuals with a diagnosis (prevalence) are subject to diagnostic evaluation. Finally, the proportion of 63% that would have tested positive with only clinical evaluation and imaging was subtracted.

In budget impact analysis, as imaging exam and clinical features are still going to be available for patients, as they are part of ASAS classification criteria for SpA, and Brazilian diagnosis recommendations, only the cost was HLA-B27 was considered.

## Results

### Systematic review

The update of the literature review retrieved 1,321 publications from the databases consulted, including 15 records. Additionally, three studies were included through manual searches, and 11 records were selected from the NICE systematic review. At the end of the process, 29 studies were included, as shown in Fig. S1 in Attachment 1. The studies had an average age that ranged from 29 to 65 years. The main characteristics of the studies and participants are presented in Tab. S2 in Attachment 1. 

Most studies were classified with “high” or “unclear” risk of bias, being penalized especially in the “patient selection” domain, due to the lack of information about the process or the use of a non-randomized/consecutive sample (Tab. S3, Attachment 1). For accuracy outcomes (sensitivity and specificity), the certainty of evidence was rated as “very low” for both comparisons of the HLA-B27 test versus ASAS and modified New York diagnostic criteria due to the risk of bias, heterogeneity among studies and indirect evidence regarding the impact on outcomes important to the patient. Details are presented in Tab. S4 and S5 in Attachment 1. 

All studies (n=29) reported the data necessary to calculate accuracy-related outcomes and were included in the accuracy meta-analyses. The HLA-B27 test was evaluated in all studies and was generally compared to the ASAS (n=10) or modified New York diagnostic criteria (n=8). The results of the individual studies are presented in Tab. S6 in Attachment 1.

**Figure 3:**
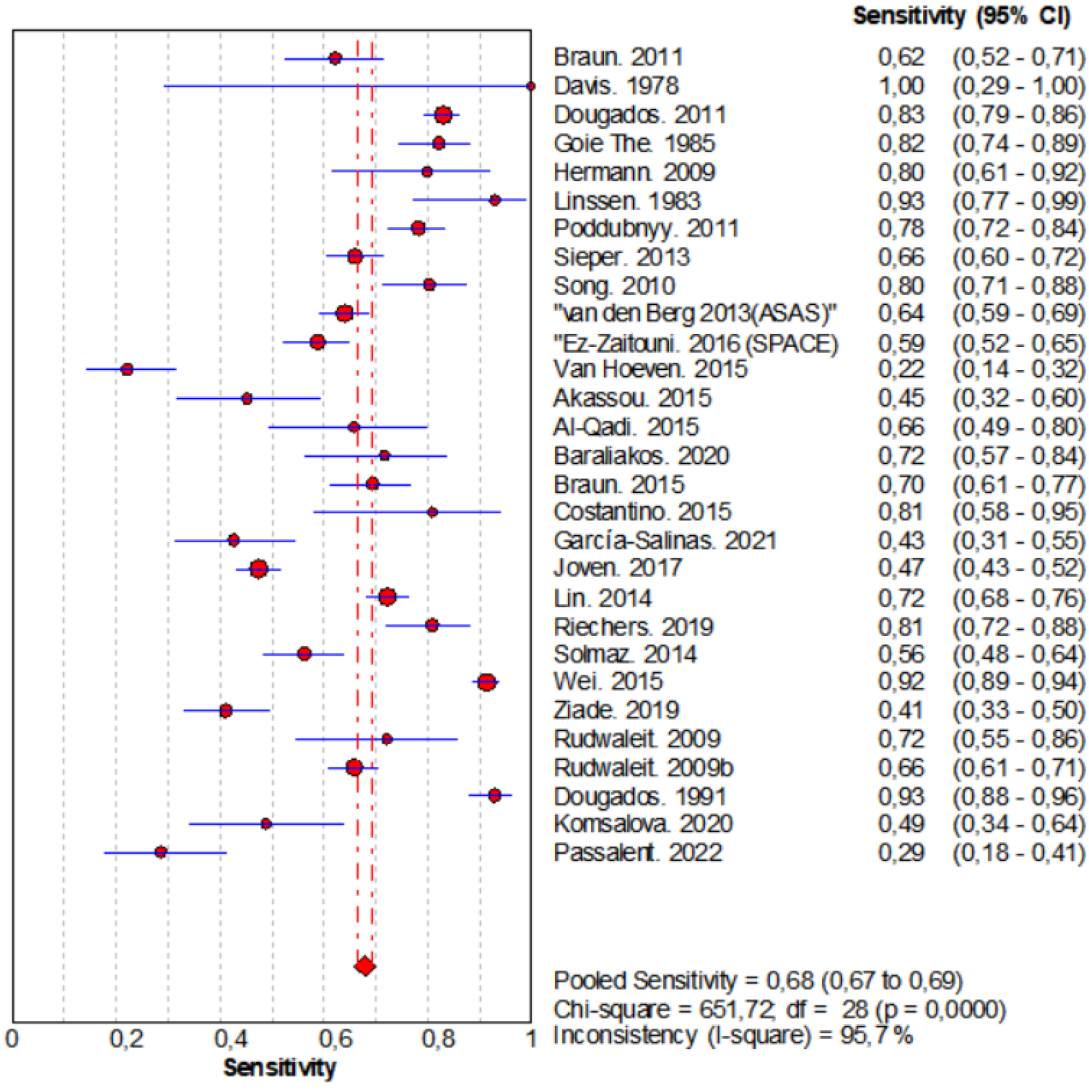
Procedure sensitivity

**Figure 4:**
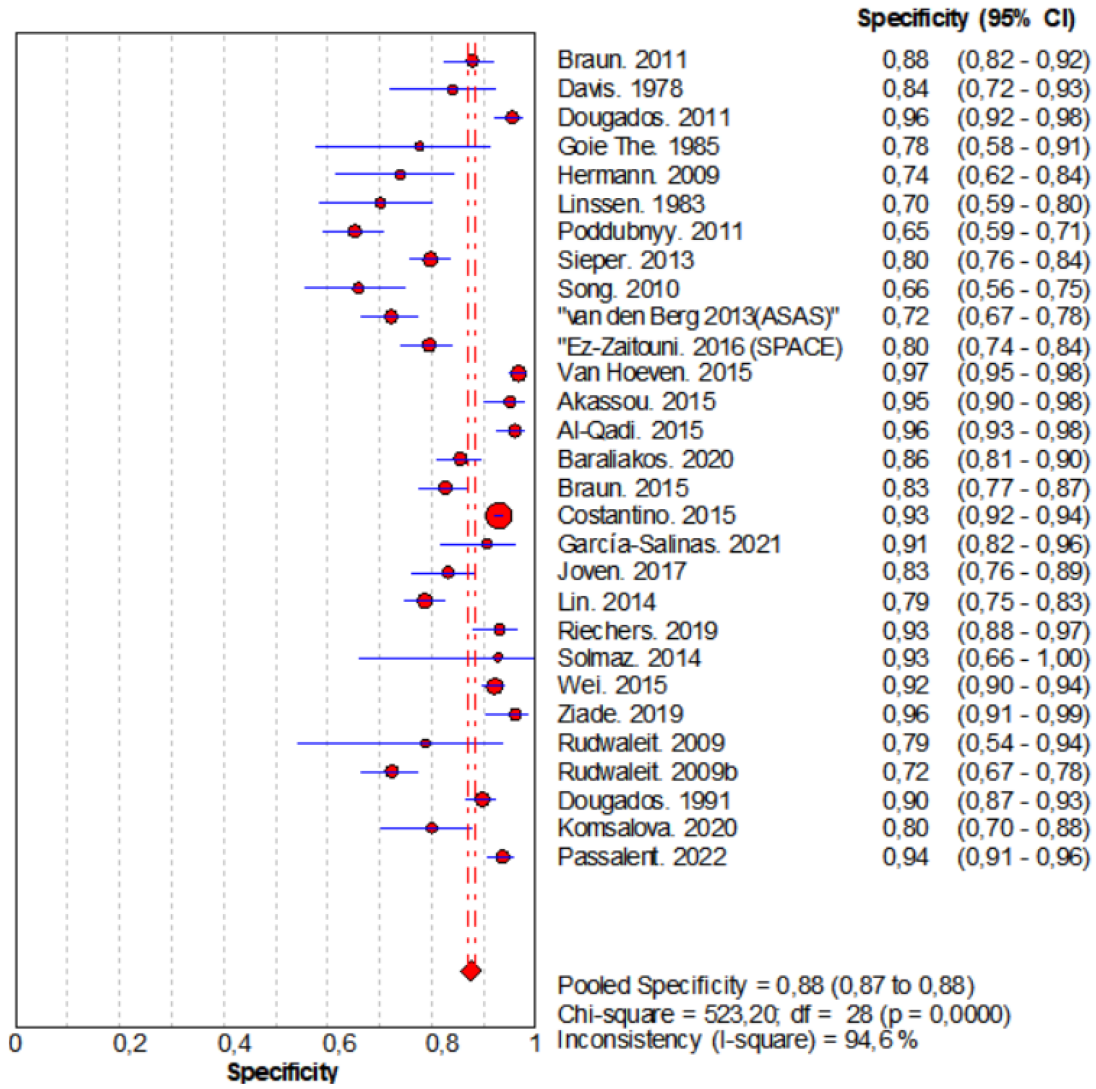
Specificity of the procedure

**Figure 5:**
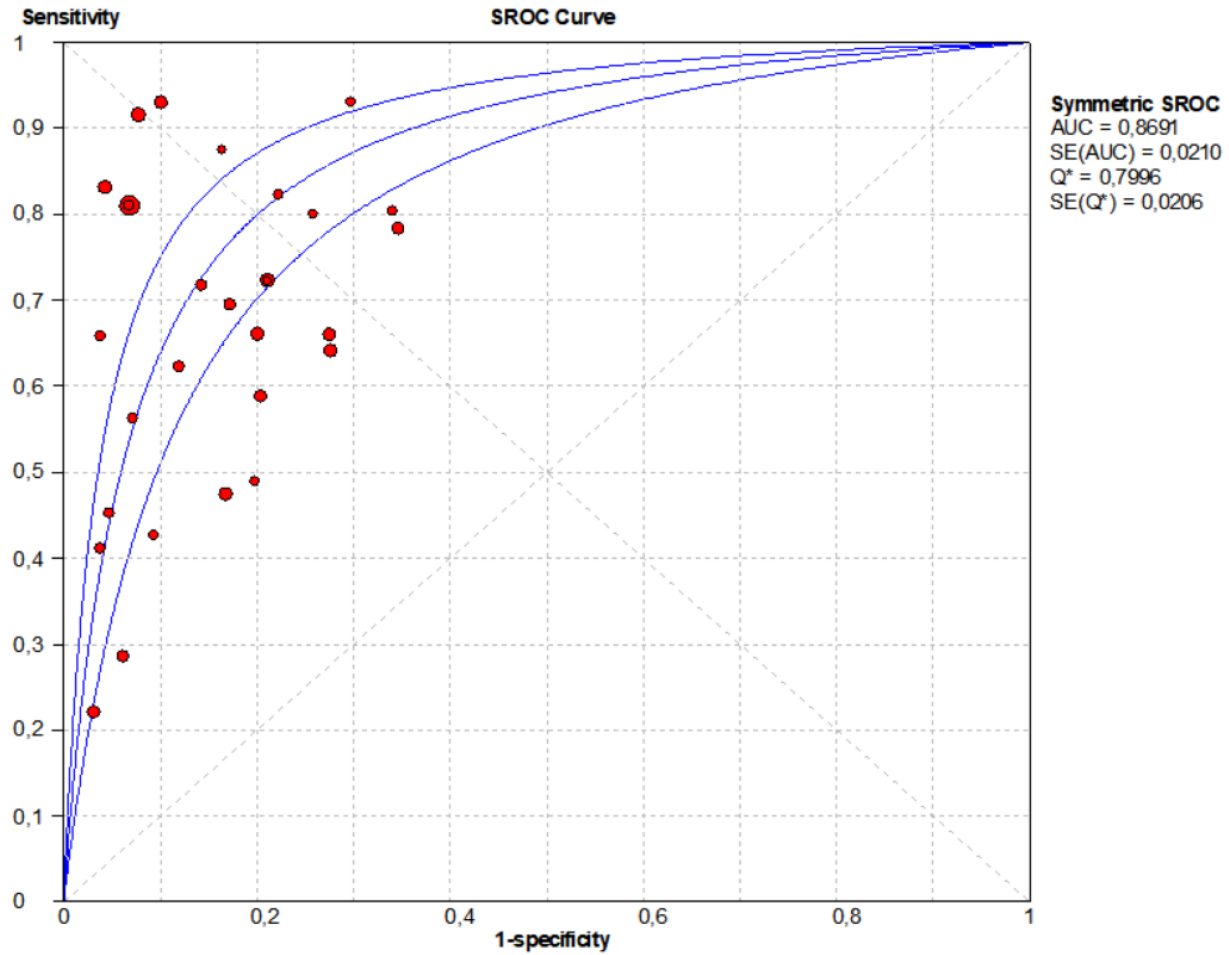
Summary ROC curve (sROC)

The estimated sensitivity for HLA-B27 was 68% (95% CI: 67-69%; Figure 3 (Fig. 3)), while the estimated specificity for HLA-B27 was 88% (95% CI: 87-88%; Figure 4 (Fig. 4)). The area under the sROC curve in the graph was 0.869 (Figure 5 (Fig. 5)).

Regarding sensitivity analyses, considering the ASAS diagnostic criteria as the reference test, the estimated sensitivity for HLA-B27 was 67% (95% CI: 65-69% and the specificity was 92% (95% CI: 91-92%; sROC 0.913). Considering the modified New York diagnostic criteria as the reference test, the estimated sensitivity of HLA-B27 was 85% (95% CI: 83-87%), and the specificity was 83% (95% CI: 81-85%; sROC: 0.859). Considering the other comparators, the estimated sensitivity for HLA-B27 was 61% (95% CI: 59-63%), and the specificity was 83% (95% CI: 81-84%; sROC: 0.845). These results are present in more detail in Fig. S2, S3 and S4 in Attachment 1.

Additionally, the study by Rudwaleit, 2009a [22] presented additional accuracy results, considering the use of HLA-B27 within the ASAS criteria in different sets of parameters. When considering the set of parameters defined by the authors, which are equivalent to the ASAS diagnostic criteria (sacroiliitis identified on radiography or MRI + at least 1 SpA feature; or at least 3 SpA features), a sensitivity of 87.7% and a specificity of 74.3% were observed. When the presence of HLA-B27 and at least two features of the disease were considered, the sensitivity was 83.3% and the specificity was 84.9%.

Rudwaleit et al. 2009b [41] also presented complementary results. Of note are the results obtained using the original criteria, a set of parameters (sacroiliitis on imaging exams—radiography or MRI + at least 1 disease feature; or at least 3 disease features), which presented a sensitivity of 97.1% and a specificity of 94.7%. Another arm presented the results for clinical evaluation (best combination of three of six disease features) and obtained a sensitivity of up to 61.1% and a specificity of up to 84.2%.

### Economic analysis 

The results of the cost-effectiveness analysis of the comparison between 'HLA-B27 + clinical evaluation' and 'clinical evaluation alone' is presented in Table 3 (Tab. 3). In the main analysis, the HLA-B27 + clinical evaluation strategy was more cost and more effective, resulting in an ICER of $17,628 per QALY.

The graph results of the probabilistic sensitivity analysis are presented in Fig. S5 in Attachment 1. Due to model uncertainties, especially regarding accuracy-related variables, the ICER of the HLA-B27 test may fall primarily into three different quadrants (32% higher cost and higher incremental effectiveness; 26% lower cost and higher effectiveness; 40% higher cost and lower incremental effectiveness).

The results of the cost-effectiveness analysis of the comparison between HLA-B27 + clinical evaluation and clinical evaluation ± imaging is presented in Table 4 (Tab. 4). In the main analysis, the HLA-B27 + clinical evaluation strategy was considered dominant, that is, it was associated with a lower cost (-$224.57) and greater effectiveness (0.02 QALY).

The graph results of the probabilistic sensitivity analysis are presented in Fig. S6 Attachment 1 . Due to model uncertainties, especially regarding accuracy-related variables, the ICER of the HLA-B27 test may fall primarily into two different quadrants (52% lower cost and higher effectiveness; 41% higher cost and lower incremental effectiveness).

### Budget impact analysis

The number of patients evaluated per year was approximately 5,000 to 7,000 new (Tab. S14 Attachment 1). Using the data from the main analysis, it was observed that incorporating the HLA-B27 test into the SUS for the proposed indication results in increased costs. The result of the analysis starts at $96,309 in the first year, reaching $116,223 in the fifth year of analysis, with an accumulated $530,393 in five years (Table 5 (Tab. 5)).

## Discussion

The findings supported the coverage of the HLA-B27 test in SUS for individuals with suspected axial spondyloarthritis, considering that test increases accuracy when associated with other diagnostic strategies and has the potential to be cost-effective or dominant. The recommendation was made by members of the Products and Procedures Committee present at the 127^th^ Regular Meeting of the National Commission for the Incorporation of Technologies into the Unified Health System (CONITEC) in March 2024. This is the committee that is responsible for advising the Ministry of Health on the incorporation, exclusion, or modification of health technologies by the SUS. The final decision to incorporate was then taken by the SECTICS Secretary (Secretariat of Science, Technology and Innovation and the Health Economic-Industrial Complex) and published in the Official Journal of the Union No. 77, section 1, page 177, on April 22, 2024. In the same context, other international Health Technology Assessment agencies have published documents that also recommend the use of HLA-B27 [45], [46].

Overall, the evidence for the primary outcomes (sensitivity and specificity) was classified as "very low” and therefore associated with a high degree of uncertainty when assessing the ability to correctly diagnose a larger number of individuals with suspected axial spondyloarthritis.

Neither way, to assess diagnostic accuracy, 29 studies were included, most of which were conducted in European countries. It is common to apply and generalize international evidence to a national context, but confounding factors may potentially be present, such as patient populations in local settings that may not be comparable to study populations, and differences in the learning curve for using technology, for example [47], [48], [49].

HLA-B27 have a positive association with family history (first-degree relatives of positive patients with the disease are more likely to develop the axial spondyloarthritis), earlier onset of disease and acute anterior uveitis, and it is known to have a strong association with spondyloarthritis [50], [51]. A previous systematic review reported that patients experienced earlier disease onset, where HLA-B27-negative patients were approximately 7-8 years older than HLA-B27-positive patients at disease onset. The risk of extra-articular manifestations, such as acute anterior uveitis may also be influenced by the HLA-B27 [52]. Besides that, the impact on faster diagnosis remains uncertain. A previous systematic review evaluating characteristics related to delay in the diagnosis of spondyloarthritis suggested that sex and family history do not appear to influence diagnostic delay. Fifteen studies evaluated HLA-B27, of which 7 indicated a reduction in diagnostic delay, 2 an increase, and 6 no differences [53]. Besides that, previous studies also showed that HLA-B27 genotype is a relevant predictor of treatment effectiveness [54], [55], [56].

In any case, delay in diagnosing spondyloarthritis can result in inappropriate expenses, including clinical evaluations, medications, and procedures [57].

Furthermore, based on the results obtained in the economic analysis, it was observed that, when compared with clinical evaluation alone, HLA-B27 + clinical evaluation achieved a result close to being considered cost-effective in the main analysis, considering the cost-effectiveness threshold of $17,628 per QALY. When compared with clinical evaluation ±imaging, HLA-B27 + clinical evaluation obtained a result considered dominant in the main analysis. However, sensitivity analyses indicated the presence of uncertainties in the model, especially regarding variables related to diagnostic accuracy.

Economic models in diagnosis area lack rigor and methodological standards, often requiring the use of assumptions that reflect the limitations of the available evidence [58], [59]. It is important to highlight that the robustness of the economic evaluation is inherently linked to the strength of the clinical and accuracy evidence previously identified. High heterogeneity introduces substantial uncertainty, which can lead to over or underestimation of the results of the economic model. In this sense, although uncertainty was tested through sensitivity analyses, results should be interpreted with caution [60].

Previous studies of economic evaluations of HLA-B27 are scarce in the literature. In this sense, the present study proposes a conceptual model that can be adapted to other countries and perspectives, recognizing the individual characteristics of each case. This may contribute to the coverage of HLA-B27 in other countries or to understanding the cost-effectiveness of this test used in certain circumstances. 

It also necessary to emphasize that the model developed was not only restricted to evaluating the short-term clinical impact, but also in the long term, following the order of disease progression.

Regarding results obtained in the BIA, it was observed that the coverage of HLA-B27 in SUS for the proposed indication would result in an average cost increase of approximately $ 106,000 per year. Additionally, especially in developing country, such as Brazil, with budgetary restrictions and limited access to certain technologies already incorporated into the public health system, conducting these analyses becomes essential to support more sustainable decisions. 

Some limitations were noted. First, considering the purpose of this study, the systematic review was mostly conducted by a single author, which may introduce selection bias. Second, some economic data were obtained through expert opinion, such as market share. Third, the reliability of the economic analysis is related to the strength of the underlying clinical and accuracy data. As the evidence was classified as “very low”, the results should be interpreted with caution.

## Conclusions

The overall body of evidence allowed us to compare the sensitivity and specificity of HLA-B27 with the ASAS or modified New York diagnostic criteria. The evidence is very uncertain about the effect of HLA-B27 on may increasing sensitivity and specificity. In economic evaluations, it was demonstrated that the exam can be cost-effective, depending on the comparator, or be close to the cost-effectiveness threshold, and will require an investment from the SUS, as observed in the budget impact analysis.

The findings presented here supported the coverage of the HLA-B27 test in the SUS, especially considering that the test increases accuracy when associated with other diagnostic strategies, and the cost-effective potential.

## Notes

### Competing interests

The authors declare that they have no competing interests (see Attachment 2).

### Funding

This work was financed by the Program to Support the Institutional Development of the Unified Health System (PROADI-SUS), Brazil.

### Authorship and contract

The author’s contract is provided in Attachment 3, and the declaration of authorship is provided in Attachment 4.

## Supplementary Material

Supplementary material

Conflict of Interests

Author’s contract

Declaration of Authorship

## Figures and Tables

**Table 1:**
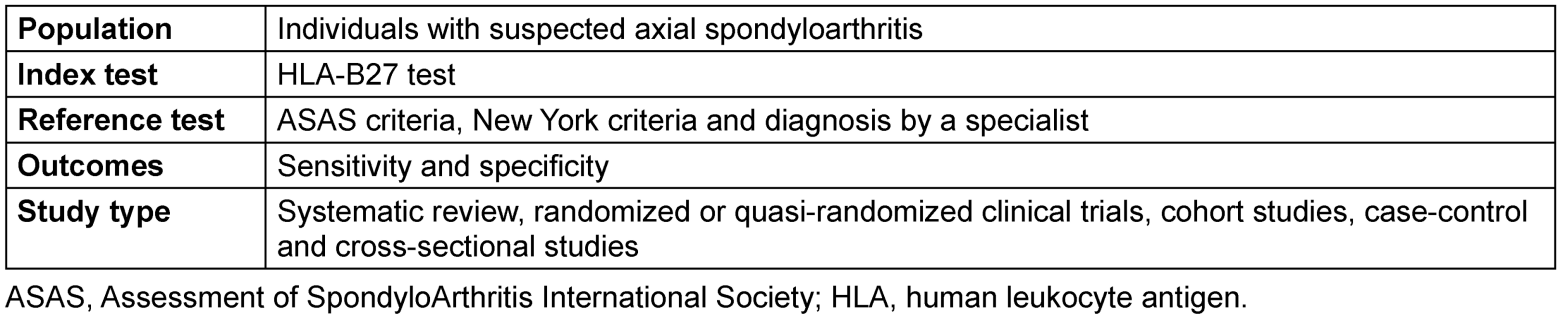
PIROS question

**Table 2:**
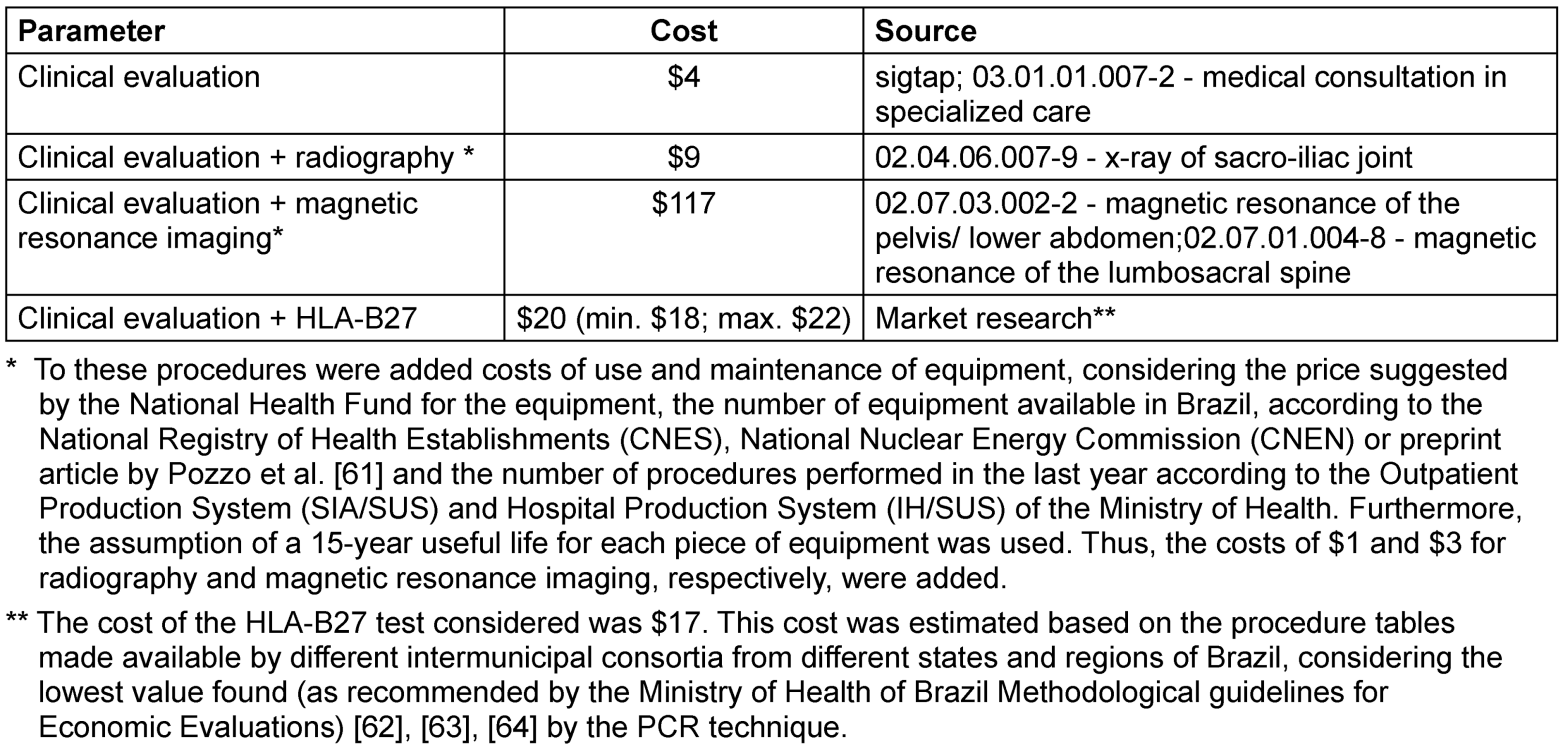
Costs considered in the main analysis

**Table 3:**
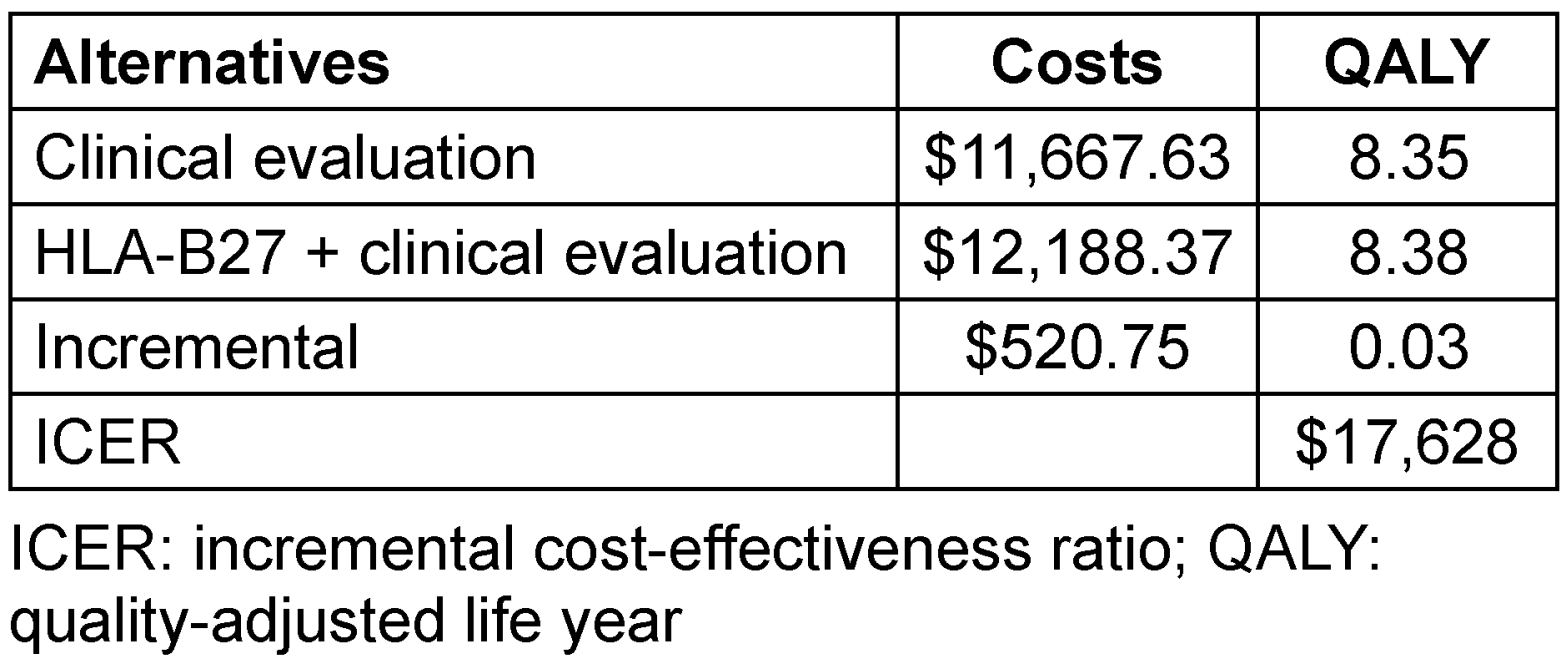
Incremental cost-effectiveness ratio table for comparing HLA-B27 + clinical evaluation versus clinical evaluation

**Table 4:**
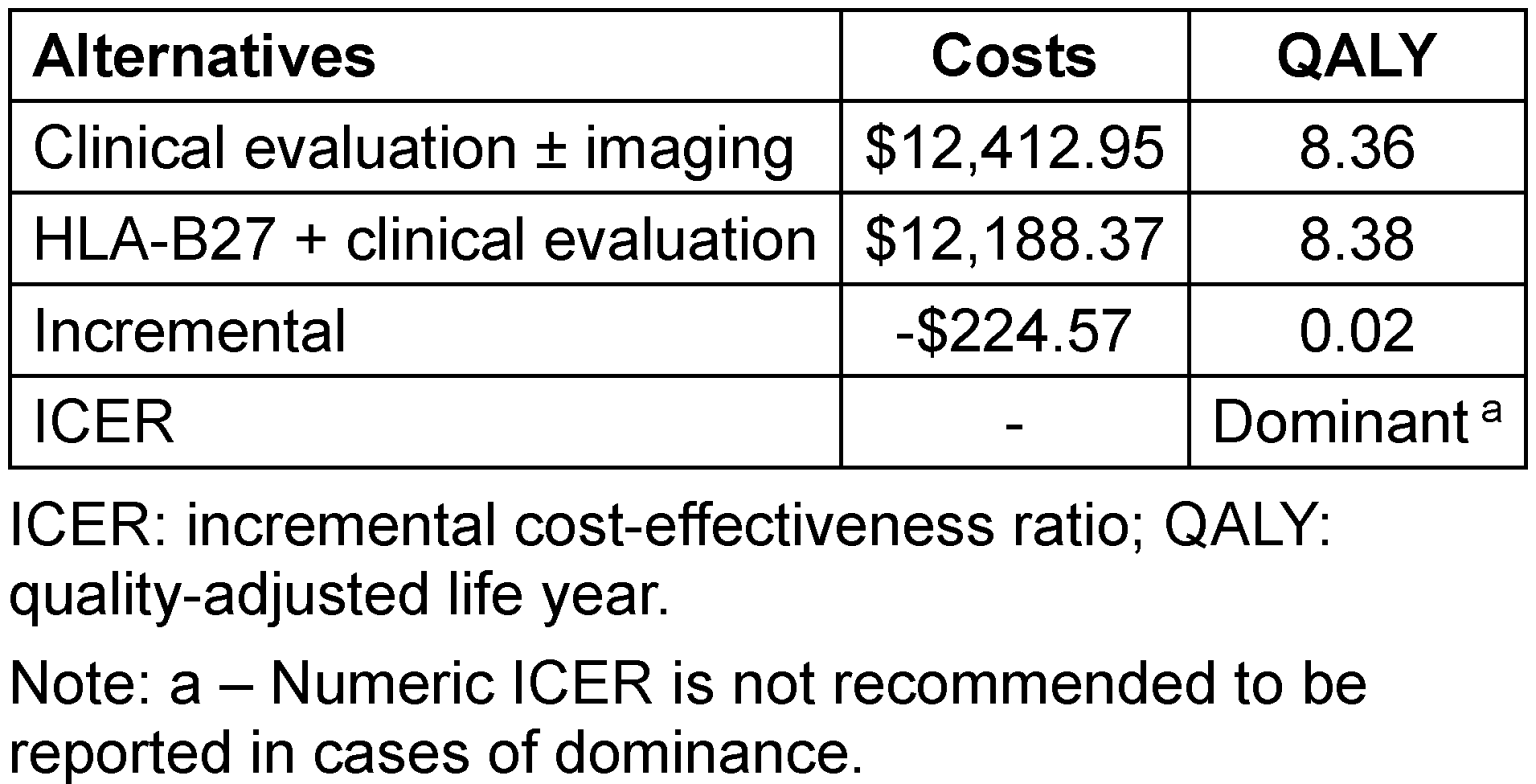
Incremental cost-effectiveness ratio table: HLA-B27 versus clinical assessment ± imaging

**Table 5:**
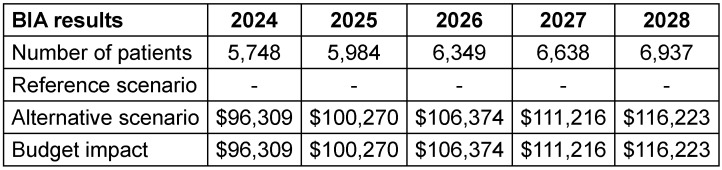
BIA results
