# Supplementary material for: HLA-B27 detection test for individuals with suspected axial spondyloarthritis to Brazilian public health system: accuracy, cost-effectiveness, and budget impact analysis
Source: GMS Health Innov Technol. 2026 Jul 1;19:Doc01. doi: 10.3205/hta000141 (PMC13366210; doi:10.3205/hta000141)
Supplement: Supplementary material [file HINT-19-01-s-001.pdf]

Supplementary material 1 - Search strategy

Database	Search strategy
**PubMed**	#1 Spondylitis, Ankylosing [MeSH] OR Ankylosing spondylitis [TIAB] OR (Ankylosing [TIAB] AND Spondy* [TIAB]) OR Spondylarthritis [TIAB] OR (Spondylitis [TIAB] AND Rheumatoid [TIAB]) OR Spondylarthritis [MeSH] OR Axial Spondyloarthritis [MeSH] OR AxSpA [TIAB] OR Non-Radiographic Axial Spondyloarthritis [MeSH] OR Spondyloarthritides [TIAB] OR "Nr-axSpA" [TIAB]<br>#2 HLA-B Antigens [TIAB] OR "HLA-B27" [TIAB] OR HLAB27 [TIAB] OR "HLA B27" [TIAB] or "HL A B27*" [TIAB] OR HLA-B27 Antigen [MeSH]<br>#3 Prognosis [TIAB] OR progression [TIAB] OR uveitis [TIAB] OR Susceptibility [TIAB]<br>#4 #1 AND #2 AND #3 (filter: date: 2014-2023)
**EMBASE**	'Ankylosing spondylitis':ab,ti OR ('Ankylosing':ab,ti AND 'Spondy*':ab,ti) OR 'Spondylarthritis':ab,ti OR ('Spondylitis':ab,ti AND 'Rheumatoid':ab,ti) OR 'AxSpA':ab,ti OR 'Spondyloarthritis':ab,ti OR 'Spondyloarthritides':ab,ti OR 'Nr-axSpA':ab,ti<br>'HLA-B27':ab,ti OR 'HLAB27':ab,ti OR 'HLA B27':ab,ti OR 'HL A B27*':ab,ti<br>'sensitiv*':ab,ti OR 'specificity':ab,ti OR 'diagnose':ab,ti OR 'diagnosed':ab,ti OR 'diagnoses':ab,ti OR 'diagnosing':ab,ti OR 'diagnosis':ab,ti OR 'diagnostic':ab,ti OR 'diagnosis':ab,ti OR 'diagnostic':ab,ti OR 'diagnostic':ab,ti OR 'accuracy':ab,ti OR ('screening':ab,ti AND 'diagnosi*':ab,ti) OR ('evidence':ab,ti OR 'assess*':ab,ti)<br>#1 AND #2 AND #3 AND [embase]/lim AND ([article]/lim OR [article in press]/lim OR [data papers]/lim OR [editorial]/lim OR [erratum]/lim OR [letter]/lim OR [note]/lim OR [review]/lim OR [short survey]/lim OR [preprint]/lim) (filter: date: 2014-2023)
**Cochrane library**	#1 "Ankylosing spondylitis":ti,ab OR ("Ankylosing":ti,ab AND "Spondy*":ti,ab) OR "Spondylarthritis":ti,ab OR ("Spondylitis":ti,ab AND "Rheumatoid":ti,ab) OR "AxSpA":ti,ab OR "Spondyloarthritis":ti,ab OR "Spondyloarthritides":ti,ab OR "Nr-axSpA":ti,ab<br>#2 "HLA-B27":ti,ab OR "HLAB27":ti,ab OR "HLA B27":ti,ab OR "HL A B27*":ti,ab<br>#3 "sensitiv*":ti,ab OR "specificity":ti,ab OR "diagnose":ti,ab OR "diagnosed":ti,ab OR "diagnoses":ti,ab OR "diagnosing":ti,ab OR "diagnosis":ti,ab OR "diagnostic":ti,ab OR "diagnosis":ti,ab OR "diagnostic":ti,ab OR "diagnostic":ti,ab OR "accuracy":ti,ab OR ("screening":ti,ab AND "diagnosi*":ti,ab) OR ("evidence":ti,ab OR "assess*":ti,ab)<br>#4 (Pubmed):an<br>#5 (#1 AND #2 AND #3) NOT #4 (filter: date: 2014-2023)

Supplementary material 2 - Values related to the diagnostic accuracy

Parameters related to diagnostic accuracy that were used individually in the models.

Parameter	Main Analysis Value (min, max)	Source
Strategy under evaluation for incorporation: HLA-B27 and at least 2 SpA characteristics		
True Positive	0.89 (0.80-0.98)	Rudwaleit, 2009 [1]
False Positive	0.11 (0.02-0.20)	Rudwaleit, 2009 [1]
True Negative	0.77 (0.69-0.85)	Rudwaleit, 2009 [1]
False Negative	0.23 (0.15-0.31)	Rudwaleit, 2009 [1]
Positivity Rate	0.56 (0.51-0.62)	Rudwaleit, 2009 [1]
Comparison available in SUS: clinical evaluation only - 3 or more SpA characteristics		
True Positive	0.85 (0.77-0.94)	Rudwaleit, 2009 [2]
False Positive	0.15 (0.06-0.23)	Rudwaleit, 2009 [2]
True Negative	0.59 (0.53-0.65)	Rudwaleit, 2009 [2]
False Negative	0.41 (0.35-0.47)	Rudwaleit, 2009 [2]
Positivity Rate	0.43 (0.39-0.47)	Rudwaleit, 2009 [2]
Comparison available in the SUS: Clinical evaluation ± imaging exam - Sacroiliitis on imaging exam and at least 1 SpA feature; or at least 3 SpA features, or 3 or more SpA features (clinical evaluation)		
True Positive	0.84 (0.75-0.92)	Rudwaleit, 2009 [1]
False Positive	0.16 (0.08-0.25)	Rudwaleit, 2009 [1]
True Negative	0.80 (0.72-0.88)	Rudwaleit, 2009 [1]
False Negative	0.20 (0.12-0.28)	Rudwaleit, 2009 [1]
Positivity Rate	0.63 (0.57-0.69)	Rudwaleit, 2009 [1]

Other probabilities used in the models

Parameter	Main Analysis Value (min, max)	Source
Probability of failure with conventional medication	0.082 (0.066;0.098)	Le, 2020 [3]
Probability of failure with biological medication (annual)	0.15 (0.12; 0.18)	Datasus (SIA-SUS)
Utility in undiagnosed individuals, true negative	0.83	Santos, 2021 [4]
Utility in undiagnosed individuals, false positive	0.78	hypothesis
Utility in responders to conventional medication	0.63	Le, 2020 [3]
Utility in non-responders to conventional medication	0.47	Le, 2020 [3]
Utility in responders to biological medication	0.57	Le, 2020 [3]
Utility in non-responders to biological medication	0.43	Le, 2020 [3]
Probability of mortality in healthy individuals (cycle 1)	0.0015	IBGE [a]
Probability of mortality in individuals with spondyloarthritis (cycle 1)	0.0018	Calculated [b]
Discount rate	0.05 (0.03; 0.10)	-

a - Data were obtained from the IBGE (Brazilian Institute of Geography and Statistics) mortality table, which provides age-specific data for all-cause mortality up to age 79.
b - Formula: P1 = healthy individuals * RR of mortality in this population. Where RR is the relative risk, i.e., 1.19 [5]. Mortality data for healthy individuals by age were obtained from the IBGE mortality table.

Direct costs details

The cost of the clinical evaluation considered was $4 (source: SIGTAP; 03.01.01.007-2 - MEDICAL CONSULTATION IN SPECIALIZED CARE. This cost was applied to this and other alternatives (that is, also to HLA-B27 and other imaging exams).

The cost of clinical evaluation + radiography (02.04.06.007-9 - SACRO-ILIAC JOINT X-RAY) was $8.7. The cost of clinical evaluation + MRI (02.07.03.002-2 - MAGNETIC RESONANCE SCANNING OF PELVIS/PELVIS/LOWER ABDOMEN; 02.07.01.004-8 - MAGNETIC RESONANCE SCANNING OF LUMBOSACRAL SPINE) was R$ 286.37. All these data were obtained from the SUS Procedures, Medications and OPM Table Management System (Sigtap). To these procedures were added costs of use and maintenance of equipment, considering the price suggested by the National Health Fund for the equipment, the number of equipment available in Brazil, according to the National Registry of Health Establishments (CNES), National Nuclear Energy Commission (CNEN) or preprint article by Pozzo

et al.[6] and the number of procedures performed in the last year according to the Ministry of Health's Outpatient Production System (SIA/SUS) and Hospital Production System (SIH/SUS). Furthermore, the assumption of a 15-year useful life for each piece of equipment was used. Thus, the costs of $1 and $3 for radiography and MRI, respectively, were added.

Based on data from scientific literature (2), data from DataSUS (Outpatient Information System - Outpatient Production, SIA-PA) and expert opinion, it was determined that most individuals would undergo only clinical evaluation or clinical evaluation and radiography (49% for the primary analysis for each, min 45%, max 49%), while the smallest portion would undergo clinical evaluation and MRI (2% for the primary evaluation, min 2%, max 10%).

The cost of the HLA-B27 test considered was $16.8. This cost was estimated based on the procedure tables provided by various intermunicipal consortia from different states and regions of Brazil, considering the lowest value found. Thus, the cost of the clinical evaluation + HLA-B27 was $20 (min 18; max 22).[7–9].

Below is information regarding the costs of other health states. The cost of the conventional treatment considered was estimated based on the cost of the medications sulfasalazine 500 mg, methotrexate 2.5 mg, and naproxen 500 mg. Based on data from DataSUS, the respective consumption proportions of these medications were estimated at 50%, 40%, and 10%. Furthermore, the annual costs were $0.4, $63, and $127, respectively (source of medication prices: Health Price Database).

For the first, second, and third biological medication statuses, the estimated annual medication consumption was based on the information presented in the Ankylosing Spondylitis PCDT, and when unavailable, in the medication package insert. This information is presented below.

Recommended dosage for biological medicines.

Drug	Dosage
**Adalimumab**	40 mg every 2 weeks
**Etanercept**	50 mg every week
**Infliximab***	Initial dose of 5 mg/kg at weeks 0, 2, and 6; followed by a maintenance dose of 5 mg/kg every 8 weeks
**Golimumab**	50 mg every 4 weeks
**Certolizumab**	Induction dose: 400 mg at weeks 0, 2, and 4; Maintenance dose: 400 mg every 4 weeks

Note: 1 week equals 7 days; Number of weeks per year: 52 weeks.
*The body weight of an adult was considered to be 70 kg for the calculation.

Based on the dosage schedule, the number of units consumed over the first and subsequent years was estimated. Costs were obtained from the Health Price Database, using the lowest value identified in the records for each medication. This information is presented in the table below.

Drugs, unit considered, costs and quantity consumed

Drug	Drug presentation	Minimum unit cost (R$) ** (main analysis)	Quantity of units consumed throughout the first year / subsequent years
Adalimumab	ADALIMUMAB, CONCENTRATION: 40 MG, PRESENTATION: INJECTABLE SOLUTION	54.8	26 / 26
Etanercept	ETANERCEPT, CONCENTRATION: 50 MG/ML, PHARMACEUTICAL FORM: INJECTABLE SOLUTION, ADDITIONAL CHARACTERISTICS: PRE-FILLED SYRINGE	116.1	52 / 52
Infliximab*	INFLIXIMAB, DOSAGE: 100 MG, PHARMACEUTICAL FORM: LYOPHILE POWDER FOR INJECTION	177.1	8 / 6
Golimumab	GOLIMUMAB, CONCENTRATION: 50 MG, PHARMACEUTICAL FORM: INJECTABLE SOLUTION, ADDITIONAL CHARACTERISTIC: IN FILLED SYRINGE, ATTACHED TO THE APPLICATION PEN	416.1	13 / 13
Certolizumab	CERTOLIZUMABE PEGOL, CONCENTRATION: 200 MG, PHARMACEUTICAL FORM: INJECTABLE SOLUTION, ADDITIONAL FEATURE: FILLED SYRINGE, WITH WET WIPES	200.5	14 / 13

*For the annual calculation, an adult's body weight was considered to be 70 kg, or 3.5 units.

**Source: Health Price Bank (BPS), consultation of the last 18 months (search date May 8, 2023), SIASG Database (Federal Purchases), administrative purchases.

The following tables show the distributions of use of biological drugs used in first-line therapy and the choices in case of treatment failure (second- or third-line). The estimated costs for these states, as well as for the "better health care" state, are presented next.

<u>Distribution of consumption of first-line biological drugs</u>

	Year 1	Year 2	Year 3	Year 4	Year 5
Adalimumab	45%	45%	45%	45%	45%
Etanercept	20%	20%	20%	20%	20%
Infliximab	10%	10%	10%	10%	10%
Golimumab	15%	15%	15%	15%	15%
Certolizumab	10%	10%	10%	10%	10%

<u>Distribution of second- and third-line treatment choices</u>

Drug	Adalimumab	Secuquinumab	Etanercept	Infliximab	Golimumab	Certolizumab
Adalimumab	-	20%	13%	6%	36%	25%
Etanercept	45%	15%	-	10%	15%	15%
Infliximab	27%	32%	14%	-	18%	9%
Golimumab	46%	22%	7%	10%	-	15%
Certolizumab	27%	27%	0%	14%	32%	-

<u>Other costs used in the model by health status</u>

Parameter	Cost used in the main analysis (min and max)	Source
Cost of the health condition "Conventional treatment" (per cycle)	$ 261 (min 235; max 287 reais)	Health Price Database (BPS) and Calculated
Cost of the health condition "First-line biological treatment" (per cycle)	$ 3588 (min 3229; max 3947)	Health Price Database (BPS) and Calculated
Cost of the health condition "Second-line or biological treatment" (per cycle)	$ 3477 (min 3129; max 3825).	Health Price Database (BPS) and Calculated

Cost of the health condition "Better health care" (per cycle)*	$ 3828 (min 3446; max 4212).	SIGTAP, Health Price Database (BPS) and Calculated

*For the state of best health care, the cost of conventional treatment, the cost of second or third line biological treatment, the cost of medical consultation, in addition to the cost of monitoring the patient in physical rehabilitation were considered (03.01.07.010-5 - INTENSIVE CARE/MONITORING OF PATIENT IN PHYSICAL REHABILITATION (1 SHIFT PATIENT-DAY - 15 CONSULTATIONS-MONTH $7 per month).

BIA - estimate the eligible population

	Year 1	Year 2	Year 3	Year 4	Year 5
Individuals with axial spondyloarthritis	7,779	8,099	8,592	8,983	9,387
Individuals with suspected axial spondyloarthritis	15,557	16,197	17,183	17,966	18,774
Individuals with suspected axial spondyloarthritis and negative imaging tests + clinical evaluation	5,748	5,984	6,349	6,638	6,937

Supplementary material 3 - Study selection flowchart and Characteristics of studies and participants.

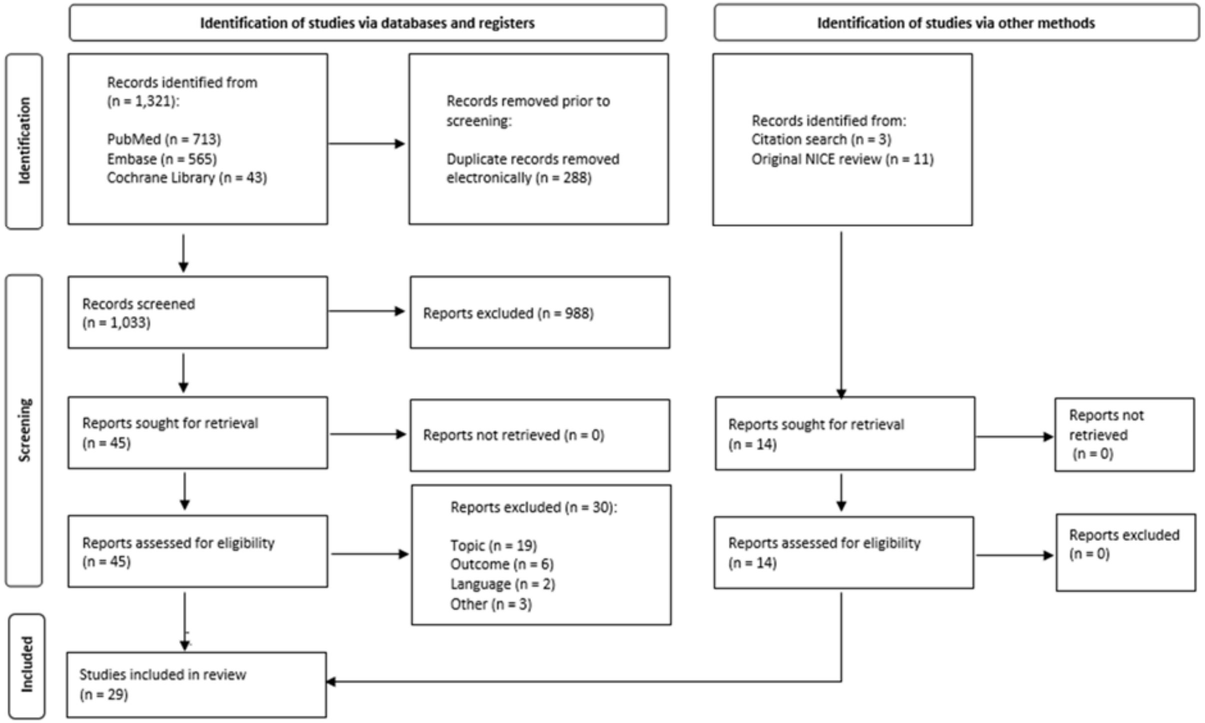

Study	Objective	Country	Patients (n)	Baseline characteristics	Reference test	Inclusion criteria
Passalent, 2022 [10]	To evaluate a stratified screening process for early identification of SpA	Canada	405	-Average age 37.<br>-45% were men.	ASAS diagnostic criteria	Patients with low back pain for more than three months and under 50 years of age.
García-Salinas, 2021 [11]	Estimate the frequency of HLA-B27 in an Argentinean SpA cohort	Argentina	150	-Average age: 44 years.<br>-43% were men.	Rheumatologists' criteria.	Patients with chronic back pain

Attachment to: Ferreira VL, Oliveira LA, Oliveira Junior JH, Lucchetta RC. HLA-B27 detection test for individuals with suspected axial spondyloarthritis to Brazilian public health system: accuracy, cost-effectiveness, and budget impact analysis. GMS Health Innov Technol. 2026;19:Doc01. DOI: 10.3205/hta000141

				-Age of onset of symptoms: 39 years		for more than 3 months.
Baraliakos, 2020 [12]	To evaluate a recently proposed two-step referral system for early recognition of SpA in primary care.	Germany	326	-Average age 35.<br>-56% were men.	ASAS diagnostic criteria and rheumatologists.	Consecutive patients with back pain and age under 45 years.
Komsalova, 2020 [13]	To analyze the predictive values of different criteria in the initial diagnosis of SpA	Spain	133	-Average age 39 years.	Modified New York and ASAS diagnostic criteria	Patients with back pain, under 50 years of age, and duration of symptoms for less than 2 years.
Riechers, 2019 [14]	To compare the sensitivity and specificity of anti-CD74 and HLA-B27 in identifying patients with non-radiographic SpA.	Germany	249	-Average age: 35.<br>-39% were men.<br>-Duration of symptoms: 16 months	ASAS diagnostic criteria	Patients aged 18-45 years, with inflammatory back pain of less than 2 years.
Ziade, 2019 [15]	To calculate the prevalence of HLA-B27 in patients with SpA compared with blood donors	Lebanon	247	-Average age 35.<br>-59% were men.	ASAS diagnostic criteria	Consecutive SpA patients and blood donors
Joven, 2017 [16]	To assess the validity of different SpA characteristics included in the Berlin and ASAS diagnostic algorithms.	Spain	665	-Average age: 33.<br>-48% were men.<br>-Duration of symptoms: 12 years	Modified New York and ASAS diagnostic criteria	National cohort composed of patients with suspected SpA.
Ez-Zaitouni, 2016 (SPACE) [17]	Investigate patients with chronic low back pain and compliance with ASAS criteria	Germany	500	- Mean age: 29 years - 37% were men - Symptom duration: 13 months	Diagnosed by rheumatologists	Patients with chronic back pain for less than 3 months and less than 2 years, aged under 45
Akassou, 2015 [18]	To determine the prevalence of HLA-B27 in healthy Moroccan controls and in patients with ankylosing spondylitis.	Morocco	181	- Mean age: 34 years - 63% were men	AMOR and ESSG criteria	Volunteers with or without SpA
Al-Qadi, 2015 [19]	To investigate the prevalence of (HLA)-B*27 among a healthy Kurdish population and in patients with ankylosing spondylitis.	Iraq	250	- Mean age: 32 years - 75% were men - Symptom duration: 7 months	Modified New York criteria	Patients with diagnosed ankylosing spondylitis and volunteers (donors)

Braun, 2015 [20]	To review the criteria for identifying young patients with SpA.	Germany	1306	- Mean age: 38 years - 49% were men - Symptom duration: 8 years	ASAS and rheumatologist criteria	Patients with chronic back pain (≥3 months) and under 45 years old
Van Hoeven, 2015 [21]	Validate and optimize a referral strategy to identify patients with chronic low back pain suspected of having SpA	Netherlands	579	- Mean age: 36 years - 41% were men<br>- Symptom duration: 7 years<br>.	ASAS criteria	Patients with chronic back pain suspected of ERA, aged 18 to 45 years, for more than 3 months
Wei, 2015 [22]	To investigate the correlation between HLA-B27 and HLA-B60 and the risk of SpA	Taiwan	1028	- Mean age: 39 years<br>–73% were men	Modified New York criteria	Patients with ankylosing spondylitis and healthy individuals
Costantino, 2015 [23]	To estimate the prevalence of SpA based on HLA-B27 in the French population.	France	6556	- Mean age: 65 years<br>- 78% were men	ASAS criteria	Functionally active French population working in electricity, aged 35 to 50 years
Lin, 2014 [24]	To evaluate the diagnostic value of ASAS classification criteria for axSpA in Chinese patients	China	867	Mean age: 29 years.<br>Symptom duration: 2 years.	ASAS diagnostic criteria	Chinese patients with chronic low back pain and non-radiographic sacroiliitis, with chronic back pain for at least 3 months
Solmaz, 2014 [25]	To evaluate the performance of new ASAS criteria for inflammatory back pain	Turkey	274	Mean age: 43 years.<br>44% were men.	ASAS diagnostic criteria	Consecutive patients with back pain or axSpA
Sieper, 2013 [26]	To determine which of two referral strategies is superior for diagnosing axSpA by rheumatologists in multiple countries	Multinational[a]	1072	Mean age: 37 years.<br>51% were men.	Diagnosis performed by rheumatologists	Patients with back pain for at least 3 months, and

						symptom onset before age 45
van den Berg, 2013 (ASAS) [27]	To compare the original Berlin algorithm to diagnose axSpA with two modifications in the SPACE cohort and ASAS criteria validation cohort	Germany	685	Mean age: 33 years.<br>43% were men.<br>Symptom duration: 8 years.	Diagnosis performed by rheumatologists	Patients without a diagnosis of chronic back pain, with back pain for at least 3 months and age below 45 years
Braun, 2011 [28]	To identify clinical predictive parameters for axSpA diagnosis in patients with chronic low back pain seen in primary care	Germany	322	Mean age: 36 years.<br>49% were men.<br>Mean age at symptom onset: 32 years.	Diagnosis performed by rheumatologists	Patients under 45 years old with back pain for more than two months
Dougados, 2011 [29]	Follow patients with early inflammatory low back pain.	France	708	-Average age: 33 years<br>-46% were men.<br>-Duration of symptoms: 18 years.	ASAS diagnostic criteria	Patients aged 18-50 years; inflammatory back pain; symptoms lasting 3 months to 3 years; and symptoms suggestive of SpA according to the local investigator's assessment.
Poddubnyy, 2011 [30]	To evaluate two referral strategies for axSpA in patients with chronic low back pain at the primary care level.	Germany	560	Mean age: 38 years.<br>54% were men.<br>Age at symptom onset: 29 years.	Diagnosis performed by rheumatologists and modified New York criteria	Patients with chronic low back pain lasting at least 3 months, under age 45, without axSpA diagnosis
Song, 2010 [31]	To assess the diagnostic value of unilateral sacroiliitis in bone scintigraphy in routine clinical practice.	Germany	207	Mean age: 40 years.<br>51% were men.<br>Disease duration: 9 years.	Modified New York diagnostic criteria	Patients with chronic low back pain attending a back pain clinic and undergoing bone scintigraphy to evaluate sacroiliitis
Hermann, 2009 [32]	To evaluate the usefulness of clinical parameters in early spondyloarthritis screening in patients	Austria	92	Group with axSpA (n=30): Mean age 32 years, 50% men, 3 years of symptoms.	Modified New York diagnostic criteria	Patients under 40 years old with back pain for at least 3

	meeting Calin criteria for inflammatory back pain.			Group with non-inflammatory back pain (n=62): Mean age 36 years, 37% men, 4 years of symptoms.		months and morning stiffness
Rudwaleit, 2009 [2]	To develop candidate classification criteria for axSpA that include patients with and without radiographic sacroiliitis.	Germany	55	Mean age: 38 years.<br>29% were men.<br>Symptom duration: 6 years.	New York criteria and rheumatologist diagnosis	Patients with chronic low back pain (unknown origin)
Rudwaleit, 2009b [1]	To validate and refine two candidate criteria sets for the classification/diagnosis of axSpA.	Multinational[b]	649	Mean age: 33 years.<br>42% were men.<br>Symptom duration: 7 years.	New York criteria and rheumatologist diagnosis	Patients with chronic low back pain (unknown origin) for at least 3 months and under 45 years old
Dougados, 1991 [33]	To define classification criteria that also include patients with undifferentiated spondyloarthritis	Six European countries	168 patients with ankylosing spondylitis and 674 in the control group	Mean age: 41 years in ankylosing spondylitis group and 50 in control group.<br>71% men in ankylosing spondylitis group and 34% in control group.<br>Disease duration: 16 years.	Rheumatologist diagnostic criteria	Patients with spondyloarthritis and controls with other rheumatic diseases
Goie The, 1985 [34]	To compare different diagnostic methods for axSpA	Switzerland	151	Not reported	Modified New York diagnostic criteria	Patients with inflammatory low back pain
Linssen, 1983 [35]	To assess the association between anterior uveitis and axSpA	Not reported	103	Mean age: 34 years for men and 45 for women.<br>56% were men.	New York diagnostic criteria	Unselected patients with uveitis
Davis, 1978 [36]	To analyze patients undergoing quantitative sacroiliitis scintigraphy and with Crohn's disease	Canada	60	Mean age: 36 years.<br>50% were men.	New York diagnostic criteria	Patients with Crohn's disease analyzed consecutively

Note: a- Canada, India, Israel, Venezuela and 12 European countries; b- Countries in Europe and Asia, plus Turkey, Canada, Colombia.

Supplementary material 4 - Risk of bias of individual studies (QUADAS-2).

	Risk of bias			
Author, year	Patient selection	Index text	Reference standard	flow and timing
Braun, 2011	X	+	-	-
Davis, 1978	-	-	-	-
Dougados, 2011	X	+	+	+
Goie The, 1985	-	+	+	+
Hermann, 2009	X	+	+	+
Linssen, 1983	-	-	-	+
Poddubnyy, 2011	+	+	-	-
Sieper, 2013	+	+	-	-
Song, 2010	X	+	+	+
van den Berg 2013	X	+	-	-
Ez-Zaitouni, 2016	X	+	-	-
Van Hoeven, 2015	+	+	+	+
Akassou, 2015	X	-	+	+
Al-Qadi, 2015	-	-	+	+
Baraliakos, 2020	+	+	-	-
Braun, 2015	-	-	+	+
Costantino, 2015	+	-	+	+

García-Salinas, 2021	-	-	-	-
Joven, 2017	X	+	-	+
Komsalova, 2020	+	+	+	+
Lin, 2014	+	+	+	+
Passalent, 2022	X	+	+	+
Riechers, 2019	-	+	+	+
Solmaz, 2014	+	-	+	+
Wei, 2015	X	-	+	+
Rudwaleit, 2009	+	+	+	+
Rudwaleit, 2009b	+	+	+	+
Dougados, 1991	-	-	+	+

GRADE assessment (comparator of ASAS diagnostic criteria)

Sensitivity	0,67 (95% CI: 0,65 to 0,69)
Specificity	0,92 (95% CI: 0,91 to 0,92)
Prevalence	0,14%, 67%,

Outucome	No. of studies (No. of patients)	Study design	Factors for evidence reduction					Effect per 1000 tested		Quality of evidence
			Risk of bias	Indirect evidence	Inconsistency	Inaccuracy	Viés de publicação	Pre-test probability of 0.14%	Pre-test probability of 45%	
**True positives**	10 studies 1543 patients	cohort and case-control studies	serious[a]	serious[b]	serious[c]	Not serious	None	1 (1 to 1)	449 (436 to 462)	⊕◯◯◯ Very low
**False negatives**								0 (0 to 0)	221 (208 to 234)	
**True negatives**	10 studies 4888 patients	cohort and case-control studies	serious[a]	serious[b]	serious[c]	Not serious	None	919 (909 to 919)	304 (300 to 304)	⊕◯◯◯ Very low
**False positives**								80 (80 to 90)	26 (26 to 30)	

A. The studies were generally classified as having a high or unclear risk of bias, particularly in the "patient selection" domain, due to the lack of information on the randomization/patient recruitment process.

B. According to the GRADE approach, diagnostic tests are usually assumed to provide indirect evidence regarding their impact on patient-important outcomes.

C. High heterogeneity was observed in the individual study effect estimates.

GRADE assessment (modified New York diagnostic criteria comparator)

Sensitivity	0.85 (95% CI: 0.83 to 0.87)
Specificity	0.83 (95% CI: 0.81 to 0.85)
Prevalence	0.14%. 67%.

Outucome	No. of studies (No. of patients)	Study design	Factors for evidence reduction					Effect per 1000 tested		Factors for evidence reduction
			Inconsistency	Inaccuracy	Inconsistency	Imprecisão	Viés de publicação	Probabilidade pré-teste de 0.14%	Probabilidade pré-teste de 45%	
**True positives**	8 studies 1091 patients		serious[a]	serious[b]	serious[c]	not serious	None	1 (1 para 1)	570 (556 para 583)	⊕◯◯◯ Very low

**False negatives**		cohort and case-control studies						0 (0 para 0)	100 (87 para 114)	
**True negatives**	8 studies<br>1265 patients	cohort and case-control studies	serious[a]	serious[b]	serious[c]	not serious	None	829 (809 para 849)	274 (267 para 281)	⊕◯◯◯<br>Very low
**False positives**								170 (150 para 190)	56 (49 para 63)	

A. The studies were generally classified as having a high or unclear risk of bias, particularly in the "patient selection" domain, due to the lack of information on the randomization/patient recruitment process.

B. According to the GRADE approach, diagnostic tests are usually assumed to provide indirect evidence regarding their impact on patient-important outcomes.

C. High heterogeneity was observed in the individual study effect estimates.

Supplementary material 5 - Results of individual studies

Study	Sensitivity	Specificity	positive predictive value	negative predictive value	Positive likehood ratio	Negative likehood ratio
Braun, 2011 [28]	0.62	0.88	0.75	0.80	5.21	0.43
Davis, 1978 [36]	1.00	0.84	0.25	1.00	6.33	0.00
Dougados, 2011 [29]	0.83	0.96	0.98	0.74	19.39	0.18
Goie The, 1985 [34]	0.82	0.78	0.94	0.49	3.70	0.23
Hermann, 2009 [32]	0.80	0.74	0.60	0.88	3.10	0.27
Linssen, 1983 [35]	0.93	0.70	0.55	0.96	3.13	0.10
Poddubnyy, 2011 [30]	0.78	0.65	0.66	0.78	2.27	0.33
Sieper, 2013 [26]	0.66	0.80	0.69	0.78	3.29	0.42
Song, 2010 [31]	0.80	0.66	0.70	0.77	2.36	0.30
van den Berg, 2013 (ASAS) [27]	0.64	0.72	0.79	0.56	2.32	0.50
Ez-Zaitouni, 2016 (SPACE) [17]	0.59	0.80	0.74	0.66	2.88	0.52
Van Hoeven, 2015 [21]	0.22	0.97	0.58	0.86	7.09	0.80
Akassou, 2015 [18]	0.45	0.95	0.80	0.81	9.66	0.57
Al-Qadi, 2015 [19]	0.66	0.96	0.77	0.93	17.20	0.36
Baraliakos, 2020 [12]	0.72	0.86	0.45	0.95	5.02	0.33
Braun, 2015 [20]	0.70	0.83	0.70	0.82	4.04	0.37
Costantino, 2015 [23]	0.80	0.93	0.09	1.00	11.67	0.21
García-Salinas, 2021 [11]	0.43	0.91	0.83	0.61	4.78	0.63
Joven, 2017 [16]	0.47	0.83	0.91	0.31	2.83	0.63
Lin, 2014 [24]	0.72	0.79	0.79	0.72	3.42	0.35
Riechers, 2019 [14]	0.81	0.93	0.89	0.88	12.07	0.20

Solmaz, 2014 [25]	0.56	0.93	0.99	0.15	7.88	0.47
Wei, 2015 [22]	0.92	0.92	0.91	0.93	11.85	0.09
Ziade, 2019 [15]	0.41	0.96	0.94	0.55	10.90	0.61
Rudwaleit, 2009 [2]	0.72	0.79	0.87	0.60	3.41	0.35
Rudwaleit, 2009b [1]	0.66	0.72	0.78	0.58	2.38	0.47
Dougados, 1991 [33]	0.93	0.90	0.76	0.97	9.21	0.08
Komsalova, 2020 [13]	0.49	0.80	0.58	0.74	2.48	0.64
Passalent, 2022 [10]	0.29	0.94	0.46	0.88	4.65	0.76

Supplementary material 6 – Subgroups analysis

Sensitivity and specificity (comparator: ASAS diagnostic criteria)

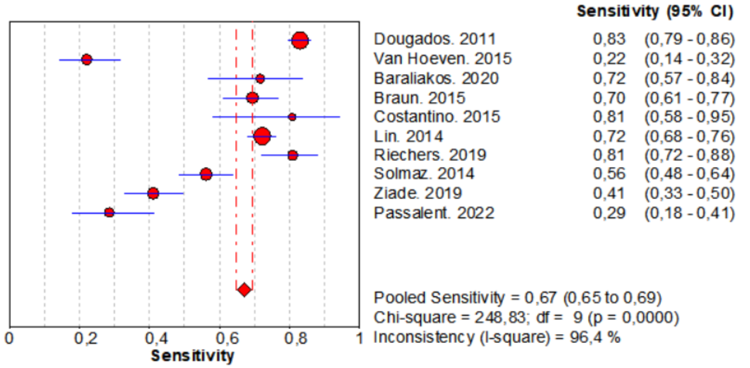

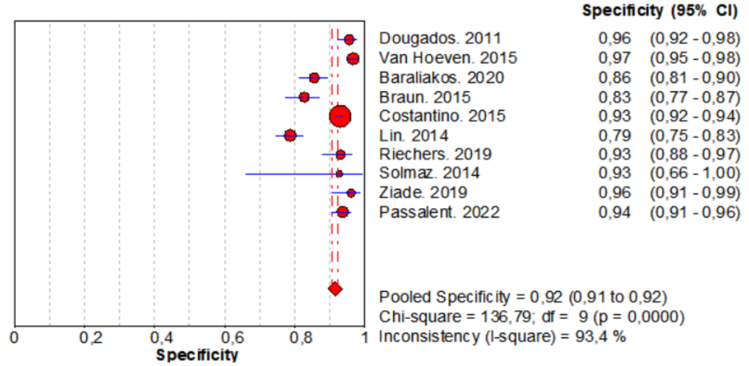

Sensitivity and specificity (comparator: modified New York diagnostic criteria)

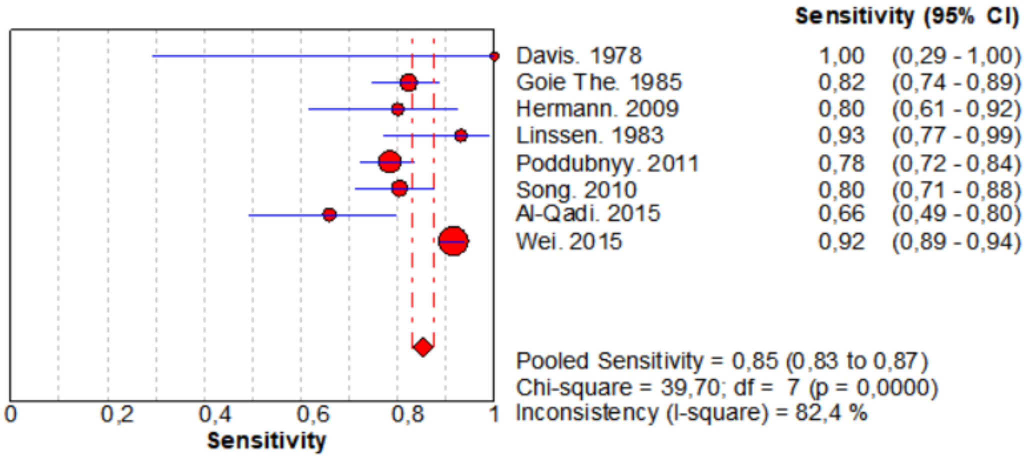

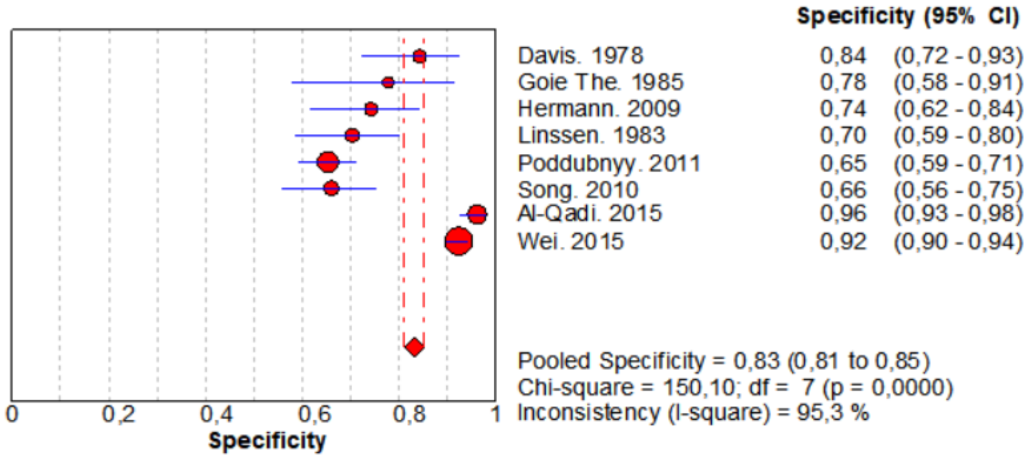

<u>Sensitivity and specificity (other comparators)</u>

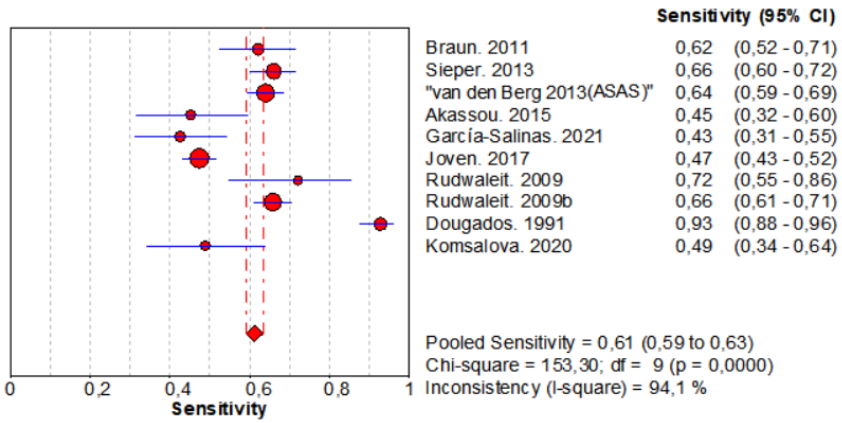

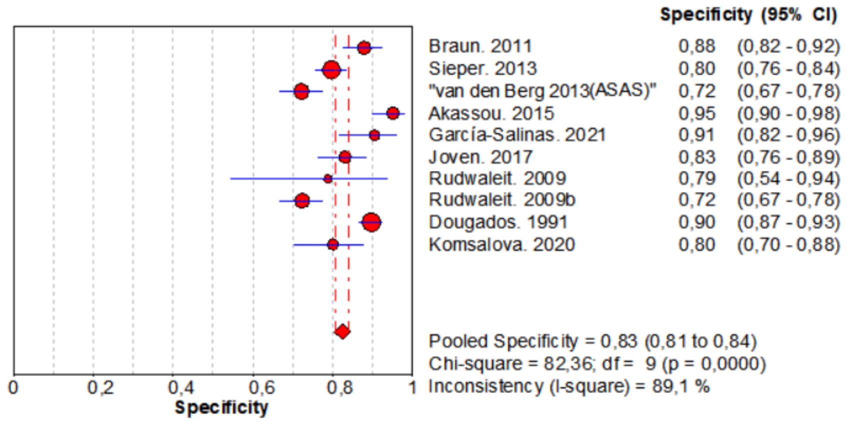

Supplementary material 7 – probabilistic sensitivity analysis

Scatterplot (HLA-B27 + clinical assessment versus clinical assessment).

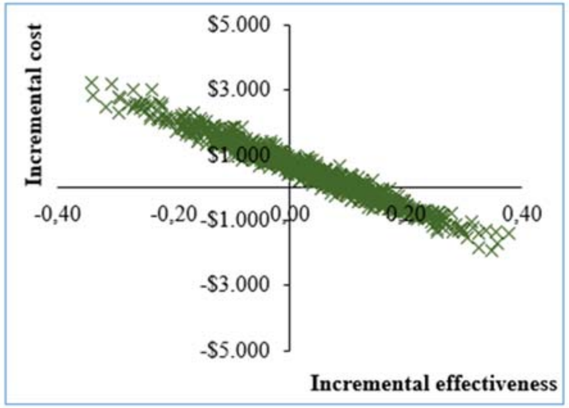

Scatterplot (HLA-B27 + clinical assessment versus clinical assessment ± imaging)

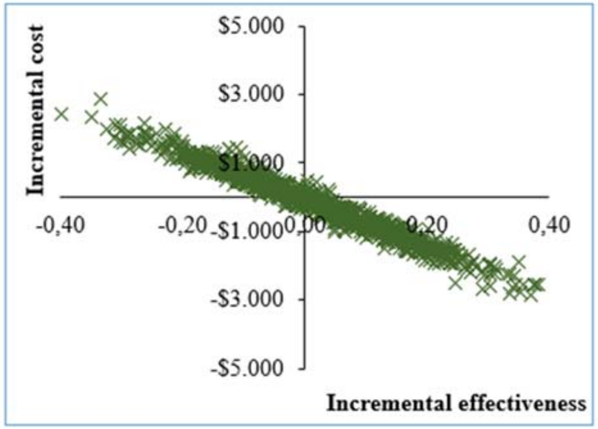

Supplementary material references

[1] Rudwaleit M, van der Heijde D, Landewé R, et al. The development of Assessment of SpondyloArthritis international Society classification criteria for axial spondyloarthritis (part II): validation and final selection. *Ann Rheum Dis* 2009; 68: 777–783.

[2] Rudwaleit M, Landewé R, van der Heijde D, et al. The development of Assessment of SpondyloArthritis international Society classification criteria for axial spondyloarthritis (part I): classification of paper patients by expert opinion including uncertainty appraisal. *Ann Rheum Dis* 2009; 68: 770–776.

[3] Le QA, Kang JH, Lee S, et al. Cost-Effectiveness of Treatment Strategies with Biologics in Accordance with Treatment Guidelines for Ankylosing Spondylitis: A Patient-Level Model. *J Manag Care Spec Pharm* 2020; 26: 1219–1231.

[4] Santos M, Monteiro AL, Santos B. EQ-5D Brazilian population norms. *Health Qual Life Outcomes* 2021; 19: 162.

[5] Ben-Shabat N, Shabat A, Watad A, et al. Mortality in Ankylosing Spondylitis According to Treatment: A Nationwide Retrospective Cohort Study of 5,900 Patients From Israel. *Arthritis Care Res (Hoboken)* 2022; 74: 1614–1622.

[6] Pozzo, L., Oliveira, M. L. de, Menezes, M. O. de, & Toscas, F. S. (2023). Nuclear Medicine in Brazilian Health System. In SciELO Preprints.

[7] ICISMEP - Instituição de Cooperação Intermunicipal do Médio Paraopeba. TABELA DE SERVIÇOS DE SAÚDE EXECUTADOS NAS UNIDADES ICISMEP. 2022.

[8] CISCENOP - Consórcio Púb Interm de Saúde do Centro. Tabelas de Valores de Procedimentos. 2019.

[9] Consórcio Intermunicipal de Saúde do Médio Paranapanema. 2022.

[10] Passalent L, Sundararajan K, Perruccio A V, et al. Bridging the Gap Between Symptom Onset and Diagnosis in Axial Spondyloarthritis. *Arthritis Care and Research* 2022; 74: 997–1005.

[11] Garciá-Salinas R, Ruta S, Chichande JT, et al. The Role of HLA-B27 in Argentinian Axial Spondyloarthritis Patients. *Journal of Clinical Rheumatology* 2022; 28: E619–E622.

[12] Baraliakos X, Tsiami S, Redeker I, et al. Early recognition of patients with axial spondyloarthritis-evaluation of referral strategies in primary care. *Rheumatology (Oxford)* 2020; 59: 3845–3852.

[13] Komsalova LY, Salinas MPM, Jiménez JFG. Predictive values of inflammatory back pain, positive HLA B27 antigen and acute and chronic magnetic resonance changes in early diagnosis of Spondyloarthritis. A study of 133 patients. *PLoS One*; 15. Epub ahead of print 2021. DOI: 10.1371/journal.pone.0244184.

[14] Riechers E, Baerlecken N, Baraliakos X, et al. Sensitivity and Specificity of Autoantibodies Against CD74 in Nonradiographic Axial Spondyloarthritis. *Arthritis Rheumatol* 2019; 71: 729–735.

[15] Ziade N, Abi Karam G, Merheb G, et al. HLA-B27 prevalence in axial spondyloarthritis patients and in blood donors in a Lebanese population: Results from a nationwide study. *International journal of rheumatic diseases* 2019; 22: 708–714.

[16] Joven BE, Navarro-Compán V, Rosas J, et al. Diagnostic Value and Validity of Early Spondyloarthritis Features: Results From a National Spanish Cohort. *Arthritis Care Res (Hoboken)* 2017; 69: 938–942.

[17] Ez-Zaitouni Z, Bakker PAC, Van Lunteren M, et al. Presence of multiple spondyloarthritis (SpA) features is important but not sufficient for a diagnosis of axial spondyloarthritis: Data from the SPondyloArthritis Caught Early (SPACE) cohort. *Annals of the rheumatic diseases* 2017; 76: 1086–1092.

[18] Akassou A, Yacoubi H, Jamil A, et al. Prevalence of HLA-B27 in Moroccan healthy subjects and patients with ankylosing spondylitis and mapping construction of several factors influencing AS diagnosis by using multiple correspondence analysis. *Rheumatol Int* 2015; 35: 1889–1894.

[19] Al-Qadi R, Salih SF, AlDoski HJ, et al. Association of HLA-B*27 with ankylosing spondylitis in Kurdish patients. *Int J Rheum Dis* 2017; 20: 980–984.

[20] Braun J, Mosch T, Fischer I, et al. [Identification of patients with axial spondylarthritis in primary care (AWARE study)]. *Z Rheumatol* 2019; 78: 568–576.

[21] van Hoeven L, Vergouwe Y, de Buck PDM, et al. External Validation of a Referral Rule for Axial Spondyloarthritis in Primary Care Patients with Chronic Low Back Pain. *PloS one* 2015; 10: e0131963.

[22] Cheng-Chung Wei J, Sung-Ching HW, Hsu YW, et al. Interaction between HLA-B60 and HLA-B27 as a better predictor of ankylosing spondylitis in a taiwanese population. *PLoS One*; 10. Epub ahead of print 2015. DOI: 10.1371/journal.pone.0137189.

[23] Costantino F, Talpin A, Said-Nahal R, et al. Prevalence of spondyloarthritis in reference to HLA-B27 in the French population: results of the GAZEL cohort. *Ann Rheum Dis* 2015; 74: 689–693.

[24] Lin Z, Liao Z, Huang J, et al. Evaluation of Assessment of Spondyloarthritis International Society classification criteria for axial spondyloarthritis in Chinese patients with chronic back pain: results of a 2-year follow-up study. *Int J Rheum Dis* 2014; 17: 782–789.

[25] Solmaz D, Akar S, Soysal O, et al. Performance of different criteria sets for inflammatory back pain in patients with axial spondyloarthritis with and without radiographic sacroiliitis. *Clin Rheumatol* 2014; 33: 1475–1479.

[26] Sieper J, Srinivasan S, Zamani O, et al. Comparison of two referral strategies for diagnosis of axial spondyloarthritis: the Recognising and Diagnosing Ankylosing Spondylitis Reliably (RADAR) study. *Ann Rheum Dis* 2013; 72: 1621–1627.

[27] van den Berg R, de Hooge M, Rudwaleit M, et al. ASAS modification of the Berlin algorithm for diagnosing axial spondyloarthritis: results from the SPondyloArthritis Caught Early (SPACE)-cohort and from the Assessment of SpondyloArthritis international Society (ASAS)-cohort. *Ann Rheum Dis* 2013; 72: 1646–1653.

[28] Braun A, Saracbasi E, Grifka J, et al. Identifying patients with axial spondyloarthritis in primary care: how useful are items indicative of inflammatory back pain? *Ann Rheum Dis* 2011; 70: 1782–1787.

[29] Dougados M, d'Agostino M-A, Benessiano J, et al. The DESIR cohort: a 10-year follow-up of early inflammatory back pain in France: study design and baseline characteristics of the 708 recruited patients. *Joint bone spine* 2011; 78: 598–603.

[30] Poddubnyy D, Vahldiek J, Spiller I, et al. Evaluation of 2 screening strategies for early identification of patients with axial spondyloarthritis in primary care. *The Journal of rheumatology* 2011; 38: 2452–2460.

[31] Song I-H, Brandt H, Rudwaleit M, et al. Limited diagnostic value of unilateral sacroiliitis in scintigraphy in assessing axial spondyloarthritis. *The Journal of rheumatology* 2010; 37: 1200–1202.

[32] Hermann J, Giessauf H, Schaffler G, et al. Early spondyloarthritis: usefulness of clinical screening. *Rheumatology (Oxford, England)* 2009; 48: 812–816.

[33] Dougados M, van der Linden S, Juhlin R, et al. The European Spondylarthropathy Study Group preliminary criteria for the classification of spondylarthropathy. *Arthritis and rheumatism* 1991; 34: 1218–1227.

[34] Goie The HS, Steven MM, van der Linden SM, et al. Evaluation of diagnostic criteria for ankylosing spondylitis: a comparison of the Rome, New York and modified New York criteria in patients with a positive clinical history screening test for ankylosing spondylitis. *British journal of rheumatology* 1985; 24: 242–249.

[35] Linssen A, Dekker-Saeys AJ, Dandrieu MR, et al. Possible ankylosing spondylitis in acute anterior uveitis. *British journal of rheumatology* 1983; 22: 137–143.

[36] Davis P, Thomson AB, Lentle BC. Quantitative sacroiliac scintigraphy in patients with Crohn's disease. *Arthritis and rheumatism* 1978; 21: 234–237.
