# Supplementary material for: HLA-B27 detection test for individuals with suspected axial spondyloarthritis to Brazilian public health system: accuracy, cost-effectiveness, and budget impact analysis
Source: GMS Health Innov Technol. 2026 Jul 1;19:Doc01. doi: 10.3205/hta000141 (PMC13366210; doi:10.3205/hta000141)
Supplement: Author’s contract [file HINT-19-01-s-003.pdf]

GMS Health Innovation and Technologies

Publisher
EuroScan international network e. V.
Editorial office
c/o dkHealth UG, Butzweilerhofallee 3
50829 Cologne, Germany

Editors-in-Chief

Dr Hong Ju
Agency for Care Effectiveness (ACE), Singapore

Dr Hans-Peter Dauben
EuroScan international network e.V. Cologne (Germany)

Author's contract GMS Health Innovation and Technologies

§ 1 Subject of the contract

1. The subject of this contract is the following submitted work by the author under the title:

   HLA-B27 DETECTION TEST FOR INDIVIDUALS WITH SUSPECTED AXIAL SPONDYLOARTHRITIS TO BRAZILIAN PUBLIC HEALTH SYSTEM: ACCURACY, COST-EFFECTIVENESS, AND BUDGET IMPACT ANALYSIS

2. The author insures that he alone is authorized to dispose over the rights of use on his work promoted by copyright and that hitherto he made no legal concessions that oppose to the provisions of this contract. This also applies to the standard texts or master illustrations supplied by the author and whose rights of use he holds. If he offers German Medical Science standard texts or master illustrations for which this does not apply, or where it is uncertain if this applies, then he has to inform German Medical Science about it and about all recognizable legally relevant facts known to him. As soon as German Medical Science assigns the author with the procurement of other standard texts or master illustrations, it requires a special agreement.
3. The author is obligated to point out to German Medical Science in writing if the work includes interpretations from persons or incidents with which the risk of an infringement of personal rights is connected. He ensures immediate notification of the service provider in case of doubt or of alleged or actual legal hindrances.
4. The rights holder exempts the service provider of any third-party claims.

§ 2 Rights

1. The author licenses to German Medical Science the non-exclusive right of duplication and circulation of machine readable data media and their safeguard media.
2. The author licenses to German Medical Science the non-exclusive right to electronic storage in databases and web pages, to make available to the public for individual requests and display on the monitor.

This also comprises the right to notify and transfer the document to third parties e. g. within the framework of national collection mandates, especially for the purpose of long-term archiving.

3. The author licenses to German Medical Science the non-exclusive right for the translation into the English language.
4. The author licenses to German Medical Science the non-exclusive right to Print-on-Demand-Production.

5. The author licenses to German Medical Science the non-exclusive right to copy and to convert the document for archiving purposes into additional, different electronic or physical formats. When doing so, impairments which are suitable to endanger the content's integrity or the intellectual and personal rights of the author on the work are to be omitted.
6. German Medical Science grants an online-access-right to the author pertaining to his work that is stored in the database.

§ 3 Contractual Obligation

1. The work will first appear exclusively in electronic form, subsequent changes of the form of the first edition require the author's agreement.
2. German Medical Science is obligated to duplicate, circulate and adequately campaign for the work in the form specified in section 1.

§ 4 Fee

1. The author(s) do not receive any honorary for their work.

§ 5 Author Designation, Copyright-Note

1. German Medical Science is obligated to appropriately identify the author as originator of the work.
2. German Medical Science is obligated to attach the copyright note with the publication of the work in the sense of the Universal Copyright Convention.
3. The author agrees that German Medical Science publishes his work under a Creative Commons Attribution 4.0 International License (http://creativecommons.org/licenses/by/4.0/). That means that anyone can copy and redistribute the material in any medium or format, provided the original work is appropriately credited. See license information at http://creativecommons.org/licenses/by/4.0/. German Medical Science is obligated to attach a note about this license upon publication of the work.

§ 6 Final Clause

If not regulated by this contract, the general legal provisions of law of the Federal Republic of Germany apply. The invalidity or inefficacy of individual regulations of this contract does not

affect the validity of the remaining regulations. The parties are then obligated to replace the deficient regulation by such a regulation whose economic and legal sense comes closest to that of the regulation to be replaced.

Brazil (São Paulo), May 11th 2026
Place, date

Signature of corresponding author
