# Supplementary material for: HLA-B27 detection test for individuals with suspected axial spondyloarthritis to Brazilian public health system: accuracy, cost-effectiveness, and budget impact analysis
Source: GMS Health Innov Technol. 2026 Jul 1;19:Doc01. doi: 10.3205/hta000141 (PMC13366210; doi:10.3205/hta000141)
Supplement: Declaration of Authorship [file HINT-19-01-s-004.pdf]

GMS Health Innovation and Technologies

Publisher
EuroScan international network e. V.
Editorial office
c/o dkHealth UG, Butzweilerhofallee 3
50829 Cologne, Germany

Editors-in-Chief

Dr Hong Ju
Agency for Care Effectiveness (ACE), Singapore

Dr Hans-Peter Dauben
EuroScan international network e.V. Cologne (Germany)

Authors/Title/ID:

Ferreira, VL; Oliveira, LA; Oliveira Junior, Haliton; Lucchetta, RC

HLA-B27 DETECTION TEST FOR INDIVIDUALS WITH SUSPECTED AXIAL SPONDYLOARTHRITIS TO BRAZILIAN PUBLIC HEALTH SYSTEM: ACCURACY, COST-EFFECTIVENESS, AND BUDGET IMPACT ANALYSIS

Declaration of Authorship

The authors declare that neither the present manuscript nor a paper with largely identical content has been published or submitted for publication.

All named authors have made a substantial contribution to this article, and all persons meeting the criteria for authorship are listed.

Brazil (São Paulo), May 11th 2026
Place, date

Signature of corresponding author

Attachment to: Ferreira VL, Oliveira LA, Oliveira Junior JH, Lucchetta RC. HLA-B27 detection test for individuals with suspected axial spondyloarthritis to Brazilian public health system: accuracy, cost-effectiveness, and budget impact analysis. GMS Health Innov Technol. 2026;19:Doc01. DOI: 10.3205/hta000141
